# New insights into morphological adaptation in common mole‐rats (*Cryptomys hottentotus hottentotus*) along an aridity gradient

**DOI:** 10.1002/ece3.11301

**Published:** 2024-04-21

**Authors:** Hana N. Merchant, Steven J. Portugal, Nigel C. Bennett, Andries K. Janse van Vuuren, Chris G. Faulkes, James Bowen, Daniel W. Hart

**Affiliations:** ^1^ Department of Biological Sciences, School of Life and Environmental Sciences Royal Holloway University of London Egham, Surrey UK; ^2^ School of Biological and Behavioural Sciences Queen Mary University of London London UK; ^3^ Department of Zoology and Entomology University of Pretoria Pretoria Gauteng South Africa; ^4^ Faculty of Science, Technology, Engineering, and Mathematics Open University Milton Keynes UK

**Keywords:** evolution, geometric morphometrics, local adaptation, shape analysis

## Abstract

Morphological adaptation is the change in the form of an organism that benefits the individual in its current habitat. Mole‐rats (family Bathyergidae), despite being subterranean, are impacted by both local and broad‐scale environmental conditions that occur above ground. Common mole‐rats (*Cryptomys hottentotus hottentotus*) present an ideal mammalian model system for the study of morphological variation in response to ecology, as this species is found along an aridity gradient and thus can be sampled from geographically non‐overlapping populations of the same species along an environmental longitudinal cline. Using the mass of five internal organs, ten skeletal measurements and 3D morphometric analyses of skulls, we assessed the morphology of wild non‐breeding individuals from five common mole‐rat populations in South Africa. We found that the body mass and mean relative mass of the spleen and kidneys in arid populations was larger, and individuals from arid regions possessed shorter legs and larger inter‐shoulder widths compared to individuals from mesic regions. Additionally, arid populations demonstrated greater skull depth, and shape change of features such as angular processes of the lower jaw than mesic individuals, indicating that these distinct geographic populations show differences corresponding to the aridity gradient, potentially in response to environmental factors such as the variation in food sources found between different habitats, in addition to different soil compositions found in the different regions. Arid populations potentially require a stronger jaw and neck musculature associated with mastication to chew xeric‐adapted plants and to dig through hard soil types, whereas mesic populations excavate through soft, looser soil and may make use of their front limbs to aid the movement of soils when digging. Aridity influences the morphology of this species and could indicate the impact of environmental changes on speciation and mammalian skull morphology.

## Graphical abstract

Distinct geographic populations of South African mole‐rats showed morphological variation that matched the predictions of the Aridity Index theory in response to factors such as the variation in food sources found in different biomes, as well as soil composition found in the different regions. Arid populations require a stronger jaw and neck musculature associated with mastication of xeric‐adapted plants, and to dig through the harder and rocky soil types. In contrast, mesic populations excavate through soft, looser soil and may make use of their front limbs to aid the movement of soil when digging. These findings are novel and suggest that ecological factors can influence the morphology within a species and could suggest the impact of environmental changes on speciation and mammalian skull morphology.

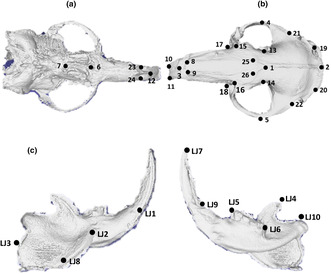

## 1 INTRODUCTION

Morphological adaptation is the change in the form and structural features of an organism that are beneficial in its current habitat (Millien et al., 2006). These morphological adaptations can be in relation to the physical environment, and can aid ecological, biological, physiological and behavioural processes. Understanding morphological changes is integral in identifying how both individuals and species respond to changes in external pressures, both in terms of evolutionary history and in more rapid response to present changes in their environment (Navas et al., 2004).

Morphology is influenced by external biotic (e.g., presence of predators and intra‐specific competition) and abiotic (e.g., water availability and temperature) adaptive forces. For example, in response to predation, specific fur colours and forms may provide camouflage. Populations of North American hog‐nosed skunks (*Conepatus leuconotus*) show intraspecific variation in colour patterns, with populations found in arid open environments having more white colourations along the dorsum compared to those populations in the forested regions. The pelage difference is believed to be a consequence of camouflage by the animals to different habitat types, as the reduced whiteness and increased black colouration in forest populations is thought to aid in camouflage in the dark understory (Ferguson et al., 2022). Morphological adaptations are more pronounced in extreme environments as a result of a greater drive for specific adaptive features. Several extreme hostile environments are found on Earth and these often require distinct adaptive features for species to survive within these ecosystems (Harris et al., 1998). Examples of this include desert and xeric regions that form the largest terrestrial biome, which in the early 21st Century covered 19% of land surface area (Lockwood et al., 2012). These arid regions can be extremely harsh and are characterised by ambient temperature extremes and seasonal or year‐round paucity of water. This presents a series of challenges for the organisms that live there, including the scarcity of resources, desiccation, heat exposure, and frequent fluctuations in temperature (Harris et al., 1998). Morphological adaptations to aridity can involve the development of exaggerated features that aid in heat dissipation such as in fennec foxes (*Vulpes zerda*), found throughout the Sahara, which possess large ears to aid temperature control and heat dissipation (Geffen & Girard, 2003). Coat colour and thickness can also facilitate heat loss in arid environments (Stuart‐Fox et al., 2017). Lighter fur morphs of springbok (*Antidorcas marsupialis*), found in the arid Karoo region of South Africa, were found to have increased heat loss when compared to dark fur morphs (Hetem et al., 2009). Furthermore, springboks were found to have thinner fur than other species of similar sizes, which is also thought to increase heat loss.

A single species may exhibit a spatial distribution across different climates, and the influence of the environment on morphology at the population level is a pivotal aspect of understanding speciation and local adaptation (Ryding et al., 2021). There is widespread evidence of population‐level changes in appendage size in response to climate change, for example, incorporating Allen's rule (Allen, 1877), which states that appendage size increases with increasing ambient environmental temperature. In addition, intra‐specific variation in body size is found to differ with climate patterns. Bergmann's rule states that populations in colder climates will have larger body sizes than those from warmer climes (Bergmann, 1847). Both Allen's rule and Bergmann's rule have been observed in many taxa (but not all) including insects, amphibians, terrestrial mammals and birds (Alho et al., 2011; Nudds & Oswald, 2007; Osorio‐Canadas et al., 2016; Symonds & Tattersall, 2010).

Currently, morphological changes at the species level in response to aridity have yet to be extensively studied, in particular, morphological changes of subterranean mammals (Ryding et al., 2021). Furthermore, different environmental conditions interact, such that aspects of morphology may often lead to adaptive trade‐offs between evolutionary pressures and co‐evolved features in response to multiple environmental stresses, for example how subterranean species cope with living underground, as well as living in arid desert conditions. African mole‐rats (Bathyergidae) are a group of subterranean rodents found across sub‐Saharan Africa including in a range of climatic regions from hyper‐mesic to hyper‐arid (Bennett & Faulkes, 2000). In addition to living in harsh arid habitats, mole‐rats occupy a subterranean niche, requiring adaptations to the small spaces of enclosed burrow systems and associated low oxygen (Bennett & Faulkes, 2000). The ability of African mole‐rats to occupy these extreme niches has been explored in species such as naked mole‐rats (*Heterocephalus glaber*), distributed in the Horn of Africa, and Damaraland mole‐rats (*Fukomys damarensis*) across the Kalahari desert (Bennett & Faulkes, 2000). It has been suggested that the ability of some African mole‐rat species to persist and thrive in arid regions of Africa is due to the adaptive benefits of group living which increases the energy allocations dedicated to foraging and locating stochastically distributed food sources. This theory, laid out by Jarvis et al. (1994) as the aridity food distribution hypothesis (AFDH) was further developed by Spinks et al. (2000), and larger individual body mass was identified in arid populations of *C. h. hottentotus* in comparison to mesic populations. Both species exhibit eusocial behaviour, which is understood to be linked to aridity through the aridity food distribution hypothesis (AFDH) (Jarvis et al., 1994). The AFDH posits that larger numbers of individuals per colony are found in arid regions to increase the chances of finding large and stochastically distributed food resources to secure sufficient energy allocations of the colony dedicated to foraging (Jarvis et al., 1994).

African mole‐rats are morphologically adapted to their fossorial lifestyle. Members of the group possess cylindrical bodies and short limbs, with large front claws, that in some species aid with movement through burrows (Bennett & Faulkes, 2000; Gomes Rodrigues et al., 2023; Jarvis, 1984; Stein, 2000). Mole‐rats are characterised by their large and powerful extrabuccal incisors which they use to dig through the soil (Jarvis, 1984). As with all rodents, these incisors continually grow throughout their life, being worn down from digging, thus enabling repeated periods of burrowing when forming their underground tunnels (Single & Dickman, 2018). Uniquely, their lips can close behind the incisors, creating a seal to prevent soil from entering the mouth as they dig (Jarvis, 1984). Because of their fossorial life, all mole‐rat species have relatively small eyes and very poor vision, only able to detect light and dark (Bennett & Faulkes, 2000; Burda, 2006). The digging strategy of *Cryptomys* species has been described as chisel‐tooth digging, whereby soil displacement is undertaken primarily by the lower incisors, while the upper incisors anchor skull to the soil (Jarvis & Sale, 1971). Chisel‐digging is found in all mole‐rat species (with the exception of those in the genus *Bathyergus*) and is linked to skull morphological features such as increased depth of skull and large upper incisor procumbence; the angle of the protrusion of the incisor from the rostrum (Lessa, 1990). This feature allows for a more favourable angle of the head for a stronger anchor in the soil and provides a greater bite force and gape to dig through harder soil types (Kraus et al., 2022; McIntosh & Cox, 2016a). Cranial morphology and digging type is thus suggested to correlate with soil hardness, and thus with habitat type (Barčiová et al., 2009).

Common mole‐rats (*Cryptomys hottentotus hottentotus*) are an ideal model species to investigate morphological variation along an environmental cline, as this mole‐rat subspecies is found in South Africa and occupies a distribution along an aridity gradient (Spinks, 1998). Unlike naked mole‐rats (*Heterocephalus glaber*) common mole‐rats possess fur. The retention of pelage and the effects on thermoregulation are not currently understood. Common mole‐rats, like all African mole‐rats, do not drink free‐standing water but rely on their food source of underground geophytes to obtain all their water requirements (Jacobs et al., 2022). Aridity has been established to influence metabolism and water uptake in mammals, with species in arid environments exhibiting lower metabolic rates and water loss than mesic‐adapted species (Tieleman et al., 2003). Oxidative stress in mesic common mole‐rat populations has been detected in the kidneys when compared to arid populations and the low levels of oxidative stress in arid populations may infer these individuals have mechanisms to combat hyperthermia and dehydration, and potentially exercise‐induced damage due to the more compact soil (Jacobs et al., 2022). Thus, precipitation and temperature, as well as the different resulting soil structure may exert differing selection pressures on individuals found in arid environments, compared to non‐arid. Common mole‐rat populations can, therefore, be used for comparisons between arid and mesic conditions operating on the same subspecies, thus, enabling the exploration of intra‐specific morphological adaptations (Bennett & Faulkes, 2000).

We aimed to explore morphological elements including body mass, body size, various measures of the limbs and feet as well as internal organ masses, and fur colour and thickness. We predicted that arid‐adapted populations would have a greater mass of organs whose function relates to water storage and filtration, thus larger organ masses of kidneys and liver. We predicted lighter fur colouration and reduced thickness of fur in arid populations compared to mesic, due to the higher temperatures and thus greater need to dissipate heat. We also predicted longer limb length, and smaller overall body size of the individuals in arid regions compared to non‐arid individuals, in accordance with Allen's and Bergmann's rules (Alhajeri et al., 2020). Additionally, as mole‐rats interact with their surroundings through their teeth, when eating and digging through soil using their incisors, we focus on the skull and the morphological aspects of the head, snout, and teeth. Arid regions will vary from non‐arid regions in the soil type and compactness, floral diversity, drier conditions and scarcity of vegetation (Naorem et al., 2023). We predict arid‐dwelling individuals to have broader snouts as well as broader zygomatic arches and coronoid processes for attachment of increased muscle mass associated with mastication due to the rocky and compact soil in the arid regions, and xeric plant types found in this habitat.

## 2 METHODS

### 2.1 Data collection

Samples of common mole rats were collected from 71 non‐breeding individuals across five sites housing discrete populations. Mole‐rat individuals were trapped using Hickman live traps (Hickman, 1979) inserted into tunnels located under mounds and were baited with sweet potato. All sites have been previously documented as having common mole‐rats (Hart et al., 2023; Spinks et al., 2000; Visser et al., 2019). Sites were specifically selected to represent an aridity gradient, based on an Aridity Index (Table 1). Aridity Index (AI) is a numerical indicator of the degree of dryness of the climate at a given location (UNEP, 1992). The AI for the study populations was calculated from climate data (ranging from the years 1981 to 2020) retrieved from the ERA5‐Land of the European Centre for Medium‐Range Weather Forecasts, created by the Copernicus Climate Change Service (Muñoz‐Sabater et al., 2021) with a spatial resolution of 0.1° by 0.1°. Monthly averaged temperature (T_air_ in °C), total precipitation (t_p_ in m), and two‐metre dew point temperature (d2m in °C) were used. These combined data were used to calculate annual aridity index (AI) (Equation (1)). Where t_p_ directly obtained from ERA5‐Land and potential evapotranspiration (PET) calculated from the Romanenko estimation (Equation (2)) (Romanenko, 1961). For Equation (2), relative humidity (RH) was calculated from ERA5‐Land d2m (Equation (3)).

(1) $$\mathrm{AI}=\frac{\mathrm{tp}}{\mathrm{PET}}$$

(2) $$\mathrm{PET}=0.00006\times \left(100–\mathrm{RH}\right)\times {\left(25+T_{\mathrm{air}}\right)}^{2}$$

(3) $$\mathrm{RH}=100\times 10^{7.591386\;\left(\frac{d2m}{d2m+240.7263}-\frac{T_{\mathrm{air}}}{T_{\mathrm{air}}+240.7263}\right)}$$

**TABLE 1:** List of 5 sites ordered from most arid to least arid, with collection coordinates for common mole rats at each site, recent Aridity Index values (from the year 2020) for each site based on climate data taken from ERA5‐Land dataset to 2 decimal places. Aridity classifications correspond to our classifications for the comparison of sites in this study.

Site	Latitude	Longitude	Aridity index	Aridity classification
Steinkopf	−29.34531	17.7872	0.04	Arid
No Heep	−30.04253	17.95852	0.07	Arid
Klawer	−31.7013476	18.7446117	0.11	Semi‐arid
Darling	−33.406573	18.417538	0.42	Mesic
Somerset West	−34.035613	18.799499	0.86	Mesic

Aridity classifications and corresponding AI values, as outlined by UNESCO (1979) and UNEP (1992) state that where PET is greater than t_p_, the climate is considered to be arid (Colantoni et al., 2015). AI values at each of the five sites used in this study are listed in Table 1 and have been selected to range in AI across different aridity classifications.

Steinkopf and No Heep were considered arid, Klawer a semi‐arid/intermediate region, whereas Darling and Somerset West are mesic. The 71 animals used in this study were maintained in captivity for 22–28 days prior to being euthanised using an overdose of isoflurane in line with strict veterinary procedures. The Animal Use and Care Committee of the University of Pretoria evaluated and approved experimental protocols (ethics clearance No. NAS016/2021) and DAFF section 20 approval (SDAH‐Epi‐21031811071). Tissues were harvested by cutting open the abdomen and chest cavity with scissors. Heart, lungs, kidney, liver, spleen, gastro‐intestinal tract, eyes and five pieces of biceps femoris muscle tissue were collected while blood samples were taken using a syringe and stored in an Eppendorf tube.

### 2.2 Age class

Age class of individuals was determined by tooth wear of molars and pattern of eruption as outlined in Bennett et al. (1990). All 71 individuals were non‐breeders and comprised a similar number of males and females in each population subsample used (Table S1).

### 2.3 Organ mass

The organs used in this study were the heart, lungs, kidneys, liver and spleen. Each organ was weighed upon extraction and stored in various solutions (Supplementary S1) according to the requirements of the experiments and studies they were subsequently used for. Mass values were recorded in mg using an electronic precision analytical weighing balance (BIOBASE, BP1003B), to the nearest 0.01 mg for 71 individuals (Steinkopf = 16, No Heep = 19, Klawer = 12, Darling = 12 and Somerset West = 12). Dissected bodies were then stored in a −80°C freezer.

### 2.4 Body measurements

Measurements were taken on defrosted bodies of 75 individuals (all 71 euthanised individuals, and an additional 4 that died of natural causes; Steinkopf = 16, No Heep = 21, Klawer = 13, Darling = 13 and Somerset West = 12) using 150 mm digital calipers (Insize, 1108) to the nearest 0.01 mm. The measurements are presented in Table 2 and depicted in Figure S1.

**TABLE 2:** List of body measurements taken (mm) from 75 common mole rats across five populations varying in Aridity Index along a gradient. Description of each measurement taken of the limbs, paws, teeth and overall body, corresponds to Figure S1 according to number.

	Measurement in mm	Measurement description
1	Body length	Tip of snout to anus
2	Inter‐shoulder width	Distance between shoulder blades
3	Inter‐hip width	Distance between hips
4	Incisor length	Incisor length top and bottom
5	Fore limb length	Heel of foot to hip/shoulder joint
6	Exposed fore paw length	Front tip of middle toe to fur line
7	Fore paw length	Tip of middle toe to heel
8	Hind limb length	Heel of foot to hip/shoulder joint
9	Exposed hind paw length	Hind tip of middle toe to fur line
10	Hind paw length	Tip of middle toe to heel

### 2.5 Pelage

Fur absorbance and reflectance were measured using Ocean Optics spectrophotometer (Ocean Optics USB2000, Oxford, UK) measuring between 329 and 1000 nm. Absorbance and reflectance were used to quantify fur colour. Following this, to measure density, each piece of fur was weighed using an electronic precision analytical weighing balance (BIOBASE, BP1003B), to the nearest mg using to measure fur thickness for each of the populations. See Supplementary S1 for full methodological details.

### 2.6 Skulls

Once body measurements were complete, skulls were extracted. See Supplementary S2 for full methodological details.

Samples were boxed and shipped on dry ice from University of Pretoria, South Africa, to Queen Mary University of London using World Courier (UK) Limited.

### 2.7 3D imaging

Skull specimens were digitised, and 3D images were created using laser surface scanning (Picza LPX‐1200DS 3D Laser Scanner). Circumferential pitch was set to 0.18 mm and height‐direction pitch was set to 0.10 mm. A preliminary scan was carried out in order to determine the resolution. Crania and right lower jaw were scanned for each sample. Left lower jaw for two individuals from each population were also scanned. Both sides of the jaw were assessed in a preliminary analysis on a subsample of the dataset to determine the magnitude of symmetry, which was found to be high (Figure S2), thus it was determined that one‐side‐only data could be used in the study (Cardini, 2016, 2017; Klingenberg et al., 2002). In total, 14 samples were omitted on account of damage to base of the skull and zygomatic arch, leaving 57 specimens left in the study (Steinkopf = 10, No Heep = 11, Klawer = 12, Darling = 12 and Somerset West = 12).

### 2.8 3D visualisation

Crania and lower jaws were digitised in 3D using MeshLab, 3D Mesh Processing System Version 2022.02 (Cignoni et al., 2008). A Screened Poisson Surface Reconstruction algorithm (Kazhdan & Hoppe, 2013) was used to build a triangulated mesh out of point cloud data for each specimen, and a PLY file was created for landmarking.

### 2.9 Landmarking

**FIGURE 1:**
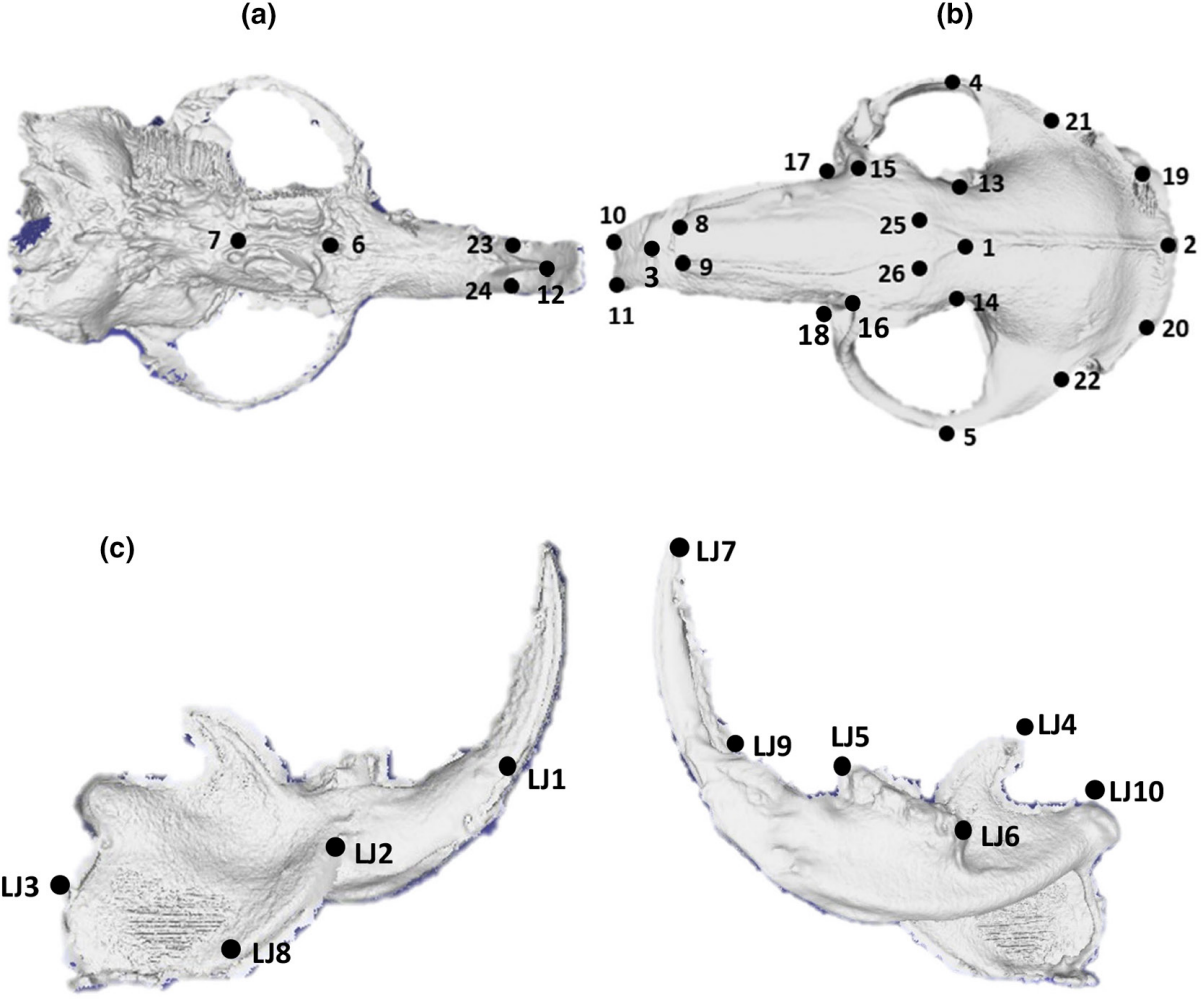
Positions of landmarks on cranium and lower jaw (LJ) of 57 *C. h. hottentotus* specimens. Landmarks are described in Table 3. (a) Dorsal view of skull, (b) ventral view of skull, (c) medial and lateral view of right lower jaw.

A configuration of 36 3D anatomical landmarks were used which were placed on forms that could be reliably and accurately located and have a clear correspondence between specimens. Focus was placed on the functional parts of the front of the skull and jaw, where the individuals would be interacting with their environment. This consisted of eight landmarks placed around the orbital region, five around the cranial base, four on the supracranium, eight around the upper dentition (incisors and cheek teeth), and ten on the lower jaw (See Table 3 and Figure 1). The cranial base is underrepresented due to the placement of the skulls during scanning, so this area has not been scanned clearly enough for landmarking. All landmarking was conducted in Checkpoint Version 2022.12.16.0419 (Stratovan Checkpoint, 2022). Five specimens were landmarked a repeat of five times to ensure intra‐observer reliability.

**TABLE 3:** Landmarks used for describing cranial and lower jaw shape in common mole rats. Landmark numbers are depicted in Figure 1. Right and left follow standard anatomical directions.

Landmark number	Landmark details
1	Intersection between inter‐parietal and inter‐frontal sutures
2	Most distal point of the supra‐occipital
3	Inter‐incisor at the pre‐maxillary joint, dorsal side
4	Outer‐most point of right zygomatic arch
5	Outer‐most point of left zygomatic arch
6	Inter‐maxillary suture (Front of top cheek teeth, between left and right cheek teeth rows)
7	Inter‐maxillary suture (Back of top cheek teeth, between left and right cheek teeth rows)
8	Intersection between the premaxillary and right nasal suture
9	Intersection between the premaxillary and left nasal suture
10	Outer corner of the tip of top right incisor
11	Outer corner of the tip of top left incisor
12	Inter‐incisor at the pre‐maxillary joint, ventral side
13	Frontal bone, inner point of right temporal fenestrae
14	Frontal bone, inner point of left temporal fenestrae
15	Intersection between the fronto‐maxillary suture‐ right
16	Intersection between the fronto‐maxillary suture‐ left
17	Intersection between the premaxillary‐maxillary suture‐ right
18	Intersection between the premaxillary‐maxillary suture‐ left
19	Lambdoid suture‐ right
20	Lambdoid suture‐ left
21	Squamosal‐zygomatic joint‐ right
22	Squamosal‐zygomatic joint‐ left
23	Top incisor at pre‐maxillary joint on outer edge‐ right
24	Top incisor at pre‐maxillary joint on outer edge‐ left
25	Intersection between the parietal and frontal suture‐ right
26	Intersection between the parietal and frontal suture‐ left
LJ1	Intersection between the incisor‐mandible‐ bottom
LJ2	Intersection between the angular process‐mandible joint
LJ3	Back of angular process, placed on the posterior
LJ4	Tip of posterior coronoid process
LJ5	Top of front molar
LJ6	Bottom of back molar at the tooth and mandible joint
LJ7	Tip of incisor
LJ8	Angular process bend
LJ9	Intersection between the incisor and mandible – top
LJ10	Anterior tip of mandibular condyle

### 2.10 Geometric morphometrics

MorphoJ Version 2.0 (Klingenberg, 2011) was used to scale, rotate, and translate the 3D landmark coordinates to carry out a Procrustes Superimposition (Ross, 2004).

### 2.11 Analyses

All statistical analyses were performed using the statistical software R version 4.2.2 (R Core Team, 2021). Principal components analyses (PCA) were conducted using the *pcrcomp* function and the factoextra package in R. PCAs were carried out on the relative mass of organs (organ mass/body mass) listed under ‘Organ mass’, to determine variation in the mass of internal organs across 71 individuals and the measurements listed in Table 2, across 75 individuals (Table S1).

Using the *glm* function, generalised linear models were used to assess population differences for the masses of each organ separately, fur reflectance, fur absorbance and fur thickness. Sex, age class, and population were included as predictor variables, with the addition of body mass for organ mass models. Sex and age class were included in the models to determine if either influence the variation in morphological characteristics. Model selection was determined using the *drop1* function and models with the highest rank were selected using Akaike Information Criterion (AIC) values, thus determining which model best explains our dataset (Grueber et al., 2011). Population and sex remained in the final models for each fur measurement, and sex, age class, body mass and population remained for the organ masses.

A PCA was also conducted on the landmarks of the crania and lower jaw separately, for 57 individuals to generate a morpho‐space of skull shape variation between the populations along an environmental gradient. using the *lda* function from the MASS package in R, a canonical discriminant analysis (CDA) (Williams, 1983) was carried out to support and highlight differences in shape between the populations. This was supported with a pairwise Mahalanobis distance matrix to quantify the distances between group means using multivariate data created using the *mahalanobis* function from the stats package in R. Finally, a pairwise nonparametric multivariate analysis of variance (NPMANOVA) was used using the *pairwise. adonis* and *p.adjust* functions from the pairwiseAdonis R package to test for a significant difference between the Procrustes coordinates, followed by a post‐hoc (pairwise) test with a Bonferroni adjustment to find potential differences between populations.

Wireframe graphs of the principal component 1 (PC1) and 2 (PC2) from the PCA analysis were generated in MorphoJ (Klingenberg, 2011) to visualise the variation in shape related to the explanatory components which included the highest percentage of shape variation across the populations.

## 3 RESULTS

### 3.1 Organ mass

**FIGURE 2:**
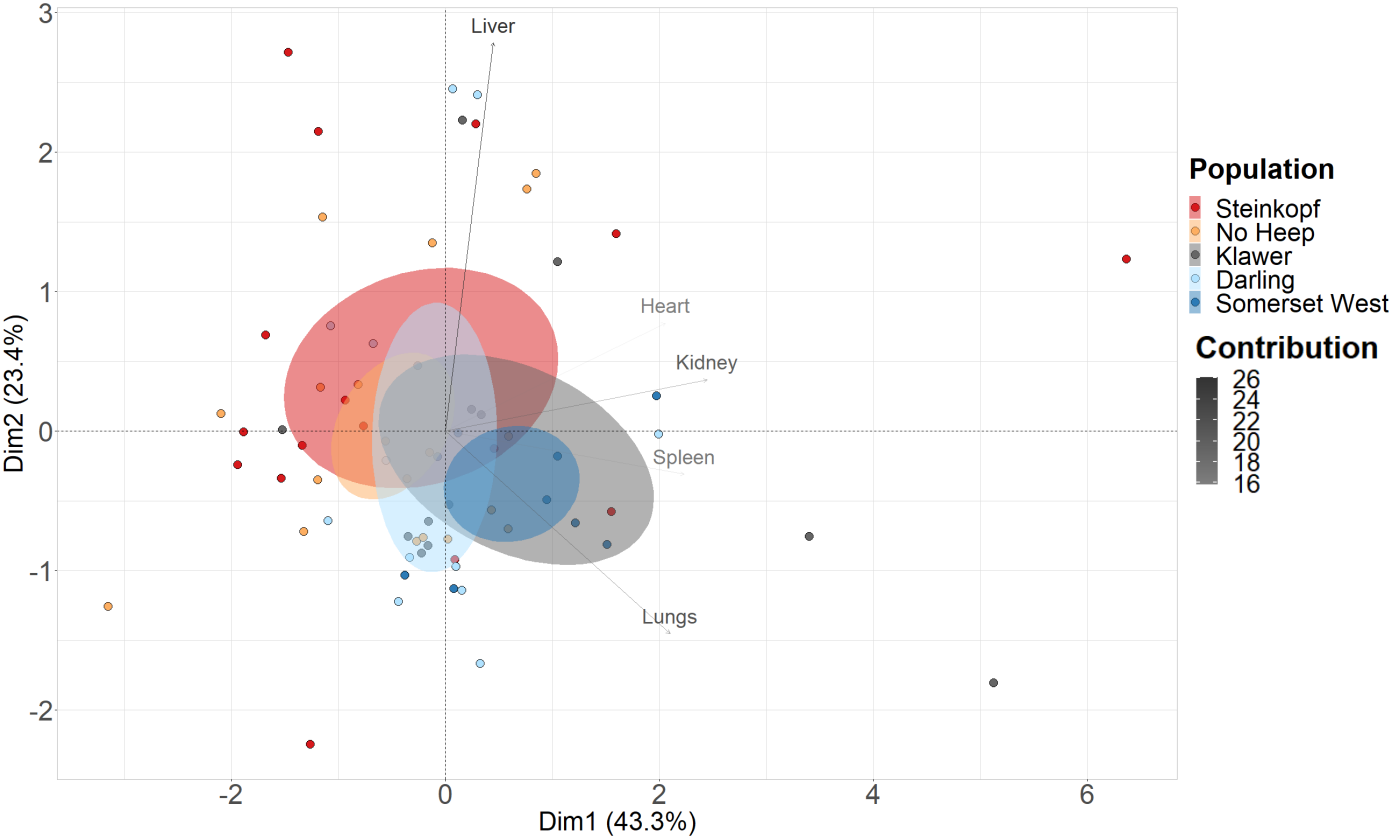
Principal component analysis based on mass corrected (relative) organ mass of 71 individuals of C*. h. hottentotus* across five populations, Steinkopf, No Heep, Klawer, Darling and Somerset West. Confidence ellipses are shaded according to population colour and define the region containing 95% of the samples drawn from the underlying Gaussian distribution. The first (Dim1) and second (Dim2) principal components display 43.3% and 23.4% of the total variation, respectively. Contributions of each variable used in the PCA analysis are displayed using a gradient, blue indicating the highest contribution and red the lowest contribution.

The PCA of relative organ mass showed that components 1–3 accounted for 81.9% of the variation in organ mass between populations (Figure 2). PC1 and PC2, displayed 43.3% and 23.4% variance (Table S3), thus, the variation exhibited between populations was well explained by the variables used in the PCA. The highest contributors were the liver, lungs and kidneys, showing the majority of differences in relative organ mass (Figure S3). Separation along the x‐axis is shown between the arid and mesic populations in the PCA. Furthermore, sex was not found to be a discriminating factor and thus variation is not linked to sex (Figure S4).

General linear models (GLMs) for each of the organs demonstrated that body mass was a significant predictor for each of the organs: heart, (GLM: *F* = 8.95, *df* = 9, 59, *p* < .001), lungs (GLM: *F* = 23.41, *df* = 9, 59, *p* < .001), kidneys (GLM: *F* = 18.01, *df* = 9, 59, *p* < .001), liver (GLM: *F* = 11.1, *df* = 9, 59, *p* < .001), spleen (GLM: *F* = 3.94, *df* = 9, 59, *p* < .001). Spleen mass was significantly higher in individuals from Somerset West (the least arid population) than all other populations.

### 3.2 Body measurements

**FIGURE 3:**
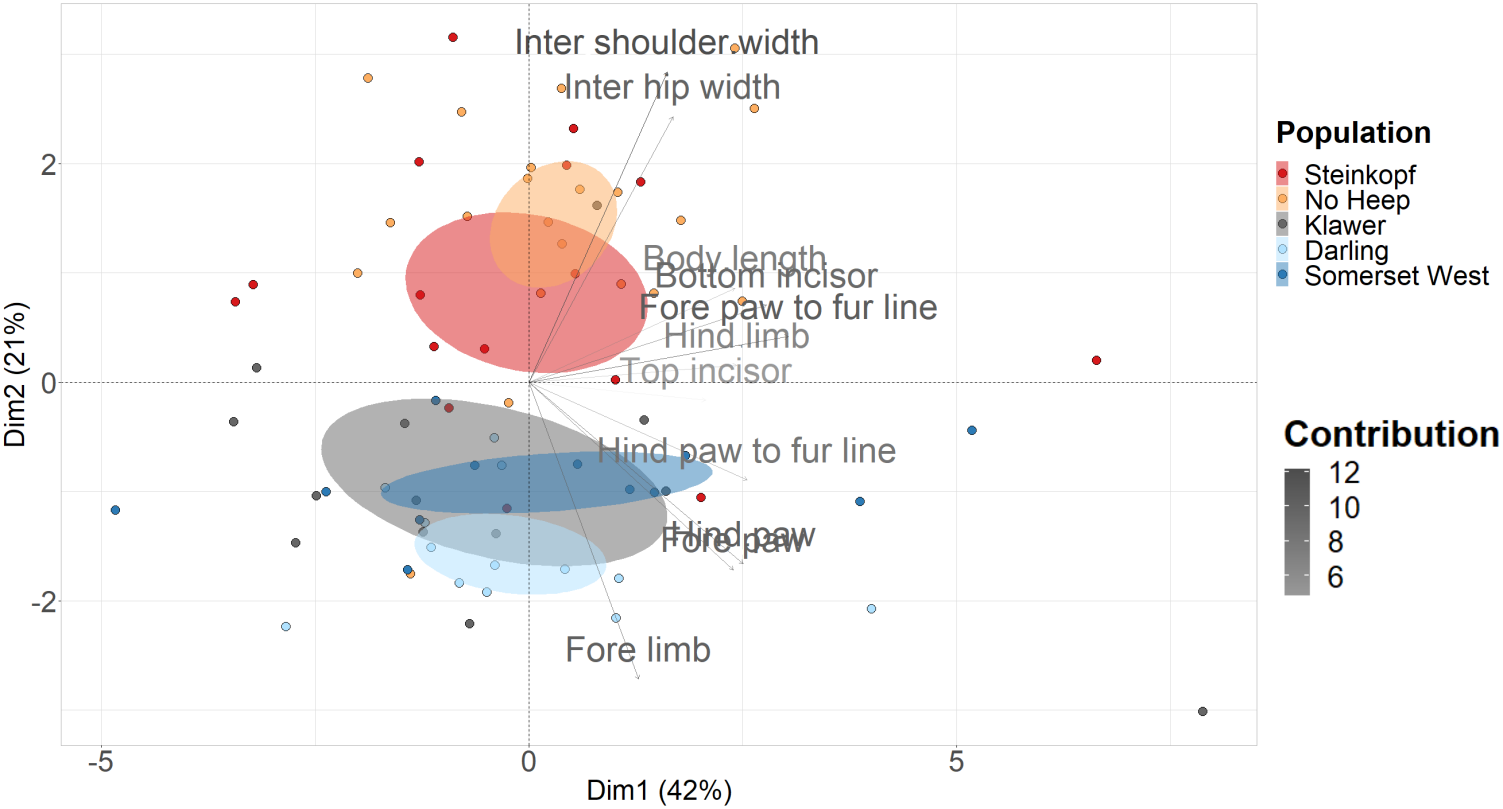
Principal component analysis based on surface skeletal body measures of 75 individuals of *C. h. hottentotus* across five populations, Steinkopf, No Heep, Klawer, Darling and Somerset West. Confidence ellipses are shaded in according to population colour and define the region containing 95% of the samples drawn from the underlying Gaussian distribution. The first and second principal component display 42% and 21% of the total variation, respectively. Contributions of each variable used in the PCA analysis are displayed using a gradient, blue indicating highest contribution and red lowest contribution.

The principal components (PC) 1–3 accounted for 70.6% of the body and surface skeletal variation. Thus, the differences between populations are well explained by the variables used in the PCA, suggesting that differences between arid and mesic populations are supported by the measurements used. PC1 and PC2 displayed 42% and 21% variance (Table S4). The highest contributors were fore limb length and inter‐shoulder width; the majority of shape variation between populations involves changes in limb length and shoulder span (Figure S5). Separation along the y‐axis is shown between the arid and mesic populations in the PCA (Figure 3). Additionally, sex was not found to be a discriminating factor and, thus, variation is not linked to sex (Figure S6).

### 3.3 Pelage

No significant difference was found between the populations in fur reflectance (GLM, *F* = 2.06, *p* = .1) and absorbance (GLM, *F* = 2.13, *p* = .09). Additionally, no difference in fur thickness was found between the populations (GLM, *F* = 1.7502, *p* = .17).

### 3.4 Morphometrics

#### 3.4.1 PCA

Principal components (PC) 1–3 accounted for 65.2% and 66% of the variation in the crania and lower jaw respectively, thus, the variables used in the PCA can moderately explain the observed differences between populations (Figure 5). PC1 and PC2 displayed 25.5% and 22.2% variance for the crania (Table S5) and 25.1% and 21.1% in the lower jaw (Table S6); the majority of shape variation involves changes in the depth of the skull and shape of the zygomatic arches (Landmarks 1, 13/14, 21/22 and 25/26). PC1 demonstrates separation between the mesic and arid populations with high intra‐group variation. Additionally, sex was not found to be a discriminating factor and thus variation is not linked to sex (Figure S7).

**FIGURE 4:**
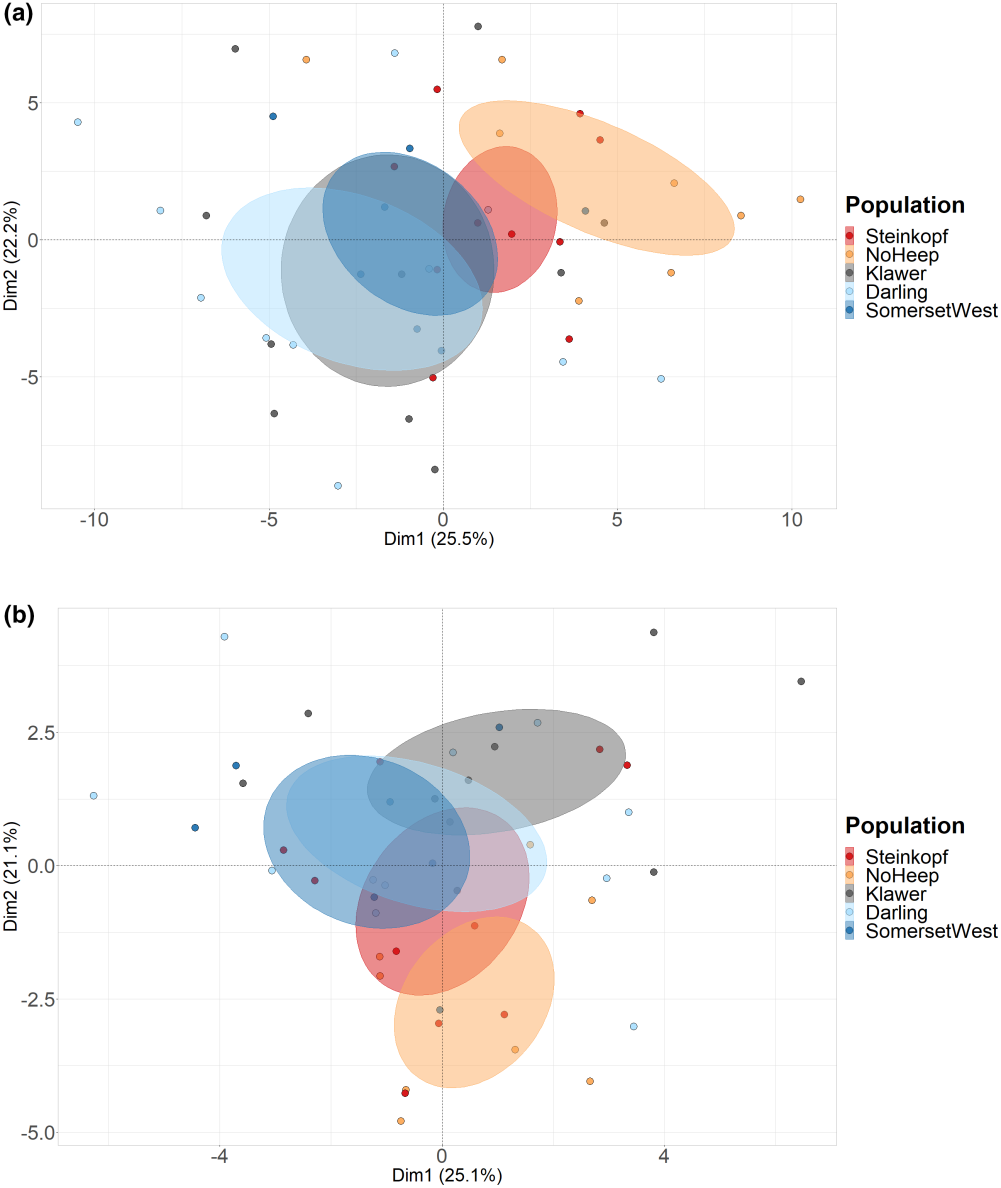
Principal component analysis based on landmark data of 57 individuals of *C. h. hottentotus* across five populations, Steinkopf, No Heep, Klawer, Darling and Somerset West for (a) crania and (b) lower jaw. Confidence ellipses are shaded in according to population colour and define the region containing 95% of the samples drawn from the underlying Gaussian distribution. The first and second principal component display 25.5% and 22.1% of the total variation, respectively for the crania, and 25.1% and 21.1% for the lower jaw.

**FIGURE 5:**
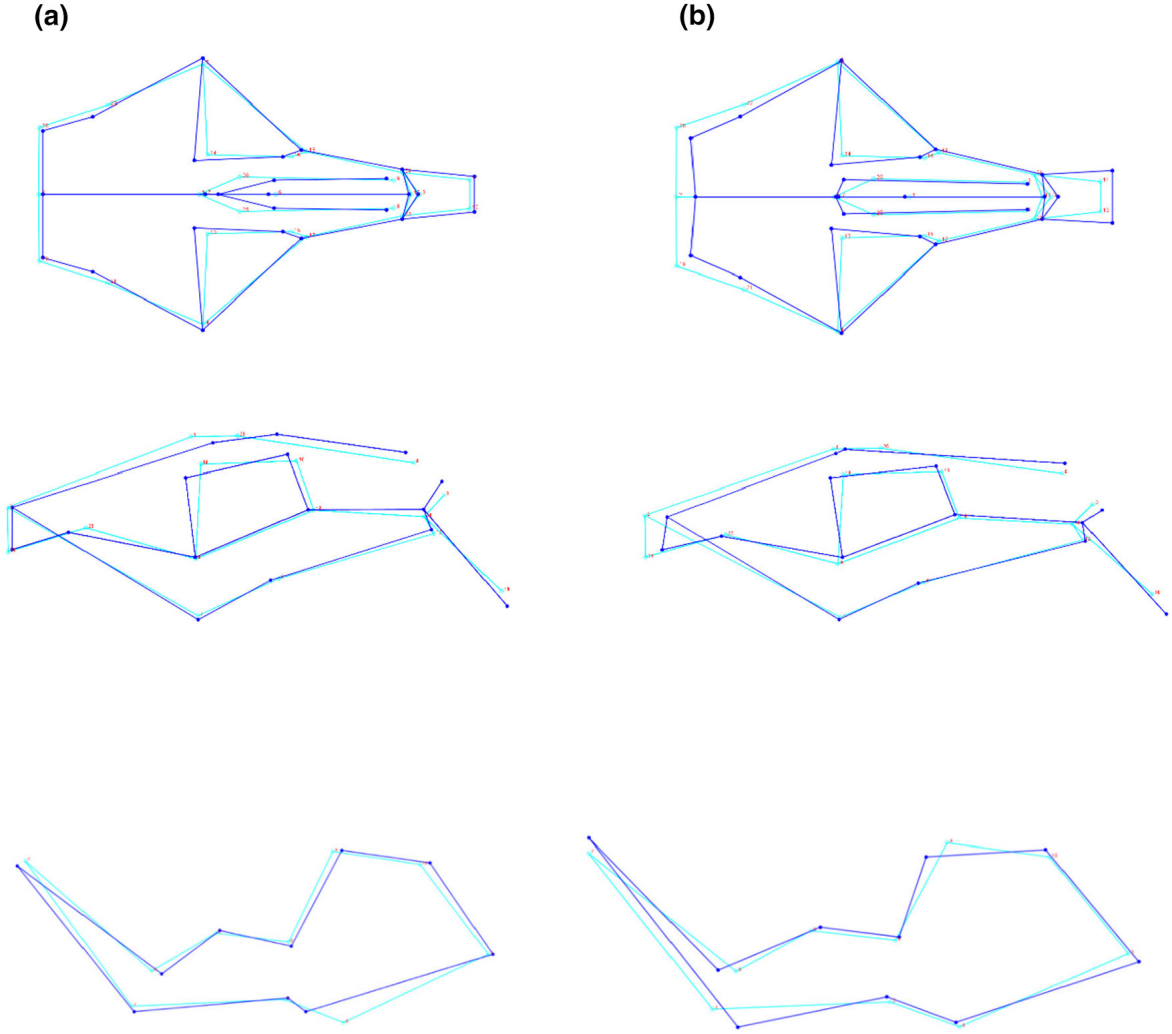
Wireframe graphs for (a) PC1 and (b) PC2 showing the average shape transformation of the crania and lower jaw (LJ) along dimension 1 from left (dark blue) to right (light blue) on the x‐axis of the PCA plot for PC1, and along dimension 2 from bottom to top on the y‐axis for PC2 (See Figure 5a).

Landmarks in PC1 deviated the most at landmarks 8 (LJ) and 1, 13/14, 21/22 and 25/26 (crania). These are where landmarks outline the zygomatic arches, dorsal side of the skull and angular process of the lower jaw. These deviations increased in size along the x‐axis of the PCA plot, indicating that more arid populations of the mole‐rats have greater dorsoventral depth of skulls and angular processes of the lower jaw that are more dorsally expanded (Figure 4). PC2 shows similar variation to PC1 in landmarks 1, 13/14, 21/22 and 25/26 (crania) indicating greater procumbence in the upper incisors. PC2 also showed an increase in size of landmarks 10/11 (crania), indicating that deviations increased in size along the axis. Additionally, there was a decrease in the distance between landmarks LJ1 and LJ9 of the lower jaw, increasing the depth of the mandible. Landmark LJ4 decreases in height (Figure 5).

### 3.5 Canonical discriminant analysis (linear discriminant analysis)

**FIGURE 6:**
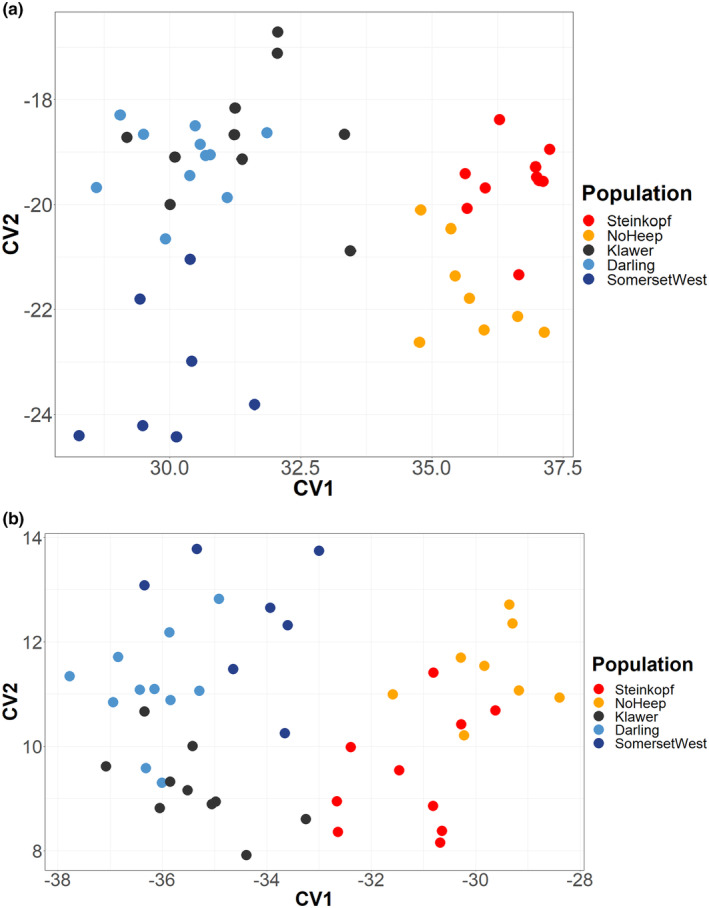
Distribution of individuals as explained by the first two canonical variates (CV1 and CV2) derived from landmark data of 57 individuals of *C. h. hottentotus* across five populations, Steinkopf, No Heep, Klawer, Darling and Somerset West for (a) the crania and (b) the lower jaw.

Canonical discriminant analysis (CDA) showed that arid and mesic populations cluster separately (Figure 6). The pairwise square Mahalanobis distance and probability values reveal that No Heep shows the greatest difference to all other populations (Table 4). Darling and Somerset West, the two least arid populations, are the most similar populations. Darling and Klawer, and Somerset West and Klawer have low values, indicating that there are similarities between these populations as well (Figures S8 and S9).

**TABLE 4:** Pairwise square Mahalanobis distance matrix among five populations of *C. h. hottentotus*. Pairwise distances are calculated from 36 landmarks across the crania (1) and lower jaw (2) of 57 specimens. All values are ×10^6^.

	Steinkopf	No Heep	Klawer	Darling	Somerset West
1					
Steinkopf	0	12.42	14.83	14.23	28.13
No Heep	12.42	0	31.53	20.83	30.85
Klawer	14.83	31.53	0	7.26	11.59
Darling	14.23	20.83	7.26	0	9.86
Somerset West	28.13	30.85	11.59	9.86	0
2					
Steinkopf	0	22.43	5.34	6.36	6.28
No Heep	22.43	0	6.71	8.71	13
Klawer	5.34	6.71	0	2.64	3.51
Darling	6.36	8.71	2.64	0	2.73
Somerset West	6.28	13	3.51	2.73	0

### 3.6 Non‐parametric pairwise MANOVA

A non‐parametric pairwise MANOVA and a post‐hoc test with a Bonferroni adjustment showed that there were no significant differences between any of the populations (NPMANOVA: *F* = 1.04, *df* = 4, *p* = .4176) for the crania and a significant difference between No Heep and Klawer only (NPMANOVA: *F* = 1.51, *df* = 4, *p* = .124) for the lower jaws (Table 5).

**TABLE 5:** Results of a post‐hoc Bonferroni adjustment of landmark data from 57 individuals of *C. h. hottentotus* populations from five populations for the crania (1) and lower jaw (2).

	df	Sum of squares	*F* model	*R* ^2^	*p* value
1					
Steinkopf vs No Heep	1	5.04	0.71	.043	.58
Steinkopf vs Klawer	1	4.42	0.72	.038	.59
Steinkopf vs Darling	1	13.03	1.66	.08	.14
Steinkopf vs Somerset West	1	3.34	0.73	.046	.58
No Heep vs Klawer	1	13.39	1.53	.088	.19
No Heep vs Darling	1	15.42	1.47	.079	.22
No Heep vs Somerset West	1	9.14	1.22	.086	.29
Klawer vs Darling	1	7.66	0.83	.042	.49
Klawer vs Somerset West	1	1.41	0.22	.015	.94
Darling vs Somerset West	1	5.6	0.67	.04	.63
2					
Steinkopf vs No Heep	1	5.096	1.54	.088	.2
Steinkopf vs Klawer	1	5.77	1.33	.069	.25
Steinkopf vs Darling	1	1.19	0.29	.015	.88
Steinkopf vs Somerset West	1	2.06	0.64	.041	.62
No Heep vs Klawer	1	11.74	3.05	.16	.03
No Heep vs Darling	1	5.71	1.57	.084	.17
No Heep vs Somerset West	1	7.15	2.94	.18	.05
Klawer vs Darling	1	5.41	1.18	.058	.29
Klawer vs Somerset West	1	10.72	2.84	.16	.04
Darling vs Somerset West	1	2.68	0.75	.045	.49

## 4 DISCUSSION

Variation in morphology and anatomy was found between populations of the common mole‐rats along an aridity gradient. The shape of the skull and body, showed variation along the AI that represents the environments where the populations were collected. Overall, a greater mass of the liver was found in arid populations of *C. h. hottentotus*, compared with those from the intermediate and mesic regions. Variations in body measurements were found between the arid populations and the intermediate and mesic, with arid populations having individuals with shorter forelimb lengths and larger inter‐shoulder widths. No significant differences in fur colour and thickness were observed between these populations. Finally, geometric morphometric analyses demonstrated variation in skull shape between mesic and arid populations. Arid populations show evidence of greater depth of skull, and shape variation in features such as angular processes of the lower jaw, and zygomatic arches, suggesting the attachment of larger masticatory muscles.

### 4.1 Organ mass

A larger liver and kidney mass was observed in the specimens derived from arid regions. The roles of the kidney include water regulation and filtration, and detoxification of substances absorbed by the digestive system (Brzoska et al., 2003). Greater kidney mass could indicate links to physiological responses to heat and dehydration such as increased water regulation and water turnover, as well as a greater ability for water retention which can be utilised during periods of stress, starvation, and dehydration (Jacobs et al., 2020, 2022). Other rodent and mole‐rat studies have explored the differences in kidney mass, size, function, and metabolism in arid and mesic regions, with similar findings (Al‐Kahtani et al., 2004; Jackson et al., 2004; Jacobs et al., 2022). Many bulbs found in the Northern Cape, where the arid populations in this study occur, have been found to be toxic, such as the bulbs of the *Drimia* plants (Manganyi et al., 2021). *Drimia* bulbs contain high levels of cardiac glycosides and are widespread across the Northern Cape (Bozorgi et al., 2017), thus, the liver and potentially the kidneys of individuals in these arid regions may be playing a role in detoxification of these food items.

A large proportion (*N* = 7 out of 12) of individuals from Somerset West showed enlarged spleens, and this could be linked to immunity as Somerset West is home to two other sympatric mole‐rat species, *Georychus capensis* (Cape mole‐rats) and *Bathyergus suillus* (Cape dune mole‐rats), (Robb et al., 2016; Thomas et al., 2013). The spleen is the primary producer of blood cells in the mammalian body, and has important functions involved in haematopoiesis (formation of blood cells such as red blood cells, macrophages and antibodies), blood filtration and immunity (Emmrich et al., 2019). An enlarged spleen is often associated with an immune response to infection or inflammation (Cheng et al., 2017). However, Cheng et al. (2017) observed that naked mole‐rats (*H. glaber*) have enlarged spleens for their size, relative to lab mice, and is believed to combat the risk of infection and disease transmission in large and tight‐knit colonies containing many individuals (Bégay et al., 2022). The anatomy of the spleen in relation to colony rank in naked mole‐rats has also been investigated whereby, enlarged spleens were shown in individuals with a higher rank within the colony (Bégay et al., 2022). Variation in spleen size has been proposed to be regulated by social interactions, and to provide immunological advantages to higher ranking individuals that patrol the colony. These individuals are also often involved in colony defence and, thus, have an increased risk of contact with predators and intruders carrying unfamiliar pathogens (Bégay et al., 2022). The individuals with enlarged spleens may be from colonies that share territories with other heterospecific species. The interaction with individuals outside of the colony may lead to exposure to foreign microorganisms, leading to enlarged spleens in individuals involved in patrol or colony protection roles. It is also possible that, by chance, individuals from higher ranks were sampled from Somerset West, compared to the arid populations, as per observations from Bégay et al. (2022).

Body mass variation was a significant influencer of organ mass, and arid dwelling individuals had a larger body mass relative to the mesic dwelling individuals. A greater body mass in arid biomes could be due to the positive relationship between size and water conservation ability (Naya et al., 2017). Arid populations are likely under selection pressures that require greater water retention to avoid dehydration, thus increased body mass. General linear models showed body mass as the only significant predictor of organ mass for all organs except the spleen.

### 4.2 Body and skull morphology

The PCA for the 10 body measurements of *C. h. hottentotus* showed differences between the arid and mesic populations; the highest contributing variables were forelimb length and inter‐shoulder width. Converse to Allen's rule, forelimbs were shorter in mole‐rats from arid populations compared to those from the mesic populations. Limb length is likely influenced by alternative selective pressures than those presented purely by climatic conditions, but further investigation into limb function and usage is needed before firm conclusions can be drawn. There is, perhaps, a trade‐off between thermoregulatory pressures of arid environments and selection for alternative traits such as locomotion and mobility in burrow systems. Several studies have shown similar patterns in limb length varying conversely to Allen's rule, across the rodent Order (Alhajeri et al., 2020), and in other subterranean and semi‐fossorial rodents (Bidau et al., 2011; Lindsay, 1987). Longer limb length is predicted in warmer climates to increase distance from the hot substrate; an adaptation that is redundant in subterranean species. Additionally, highly adaptive features are required for mobility in burrows or shelters, thus fossoriality may be a stronger influencer on limb length than aridity. In the mesic regions, *Bathyergus suillus* is found sympatrically with common mole‐rats, this species utilises claw digging as well, to facilitate digging with their teeth (Bennett & Faulkes, 2000). Soft, sandy soils are found in the mesic regions and as such, burrows will collapse when digging, so *B. suillus* needs the ability to move soil out of the way with their feet as they dig. The longer limb length found in the mesic populations suggest that these populations could be tending towards utilising some degree of forelimb movement to facilitate digging in soft soils. Indeed, front paw length was the fourth highest contributor. Montoya‐Sanhueza et al. (2022) found that solitary species of African mole‐rats exhibit increased limb bone specialisation compared to social species, due to the shared costs of digging in colonial species. This may further suggest that arid populations of common mole‐rats are living in larger colonies, and that digging roles are shared between a larger number of individuals.

Cranial and lower jaw shape varies along the environmental gradient and shows evidence of separate morphotypes in arid and mesic habitats (Barčiová et al., 2009). Arid populations demonstrated skull shape changes linked to increased muscle attachment, such as broader rostra, wider zygomatic arches, larger temporal fossae and greater depth of the skull. Furthermore, the arid individuals also had a larger inter‐shoulder width than mesic individuals. The harder soil found in arid regions necessitates stronger muscles to loosen and remove the soil (Kraus et al., 2022). The difference in inter‐shoulder width may be linked to neck and chest muscle attachments of the different populations and the increased muscle mass of the skull may require greater muscular and skeletal anatomy of the skull and shoulders for support. The greater inter‐shoulder width of individuals in arid populations also suggests increased muscle mass potentially needed for the forelimbs to aid in movement through harder‐packed soil. The AFDH suggests increased demand for foraging due to sparse geophyte distribution in arid populations of common mole‐rats which may require increased digging compared to the mesic populations to find food, therefore requiring larger muscles for this constant foraging. Evidence exists for more extensive tunnel systems in the arid populations compared to the mesic populations (Spinks et al., 2000), and this may necessitate larger skull features that support masticatory muscle attachments, for the greater muscular mass. This suggests increased muscle mass indicates increased strength to dig and extend the tunnel systems. Mammalian masticatory morphology is known to be a highly plastic region of the skull. The functional morphology of crania and masticatory musculature in several mammalian species has been studied in relation to ecological factors such as diet, habitat, locomotory and activity patterns and found to be linked to diet and bite force (Gomes Rodrigues et al., 2016; Gomes Rodrigues & Damette, 2023). This indicates that larger muscle masses are related to greater bite force and increased chewing, particularly regarding dentition and the lower jaw (Becerra et al., 2011; Borges et al., 2017; Gomes Rodrigues et al., 2023). Hystricognath rodents have shown variations in morphology associated with mastication in line with variations in habitat and diet (Hautier et al., 2012). Similar patterns have been found in other species such as punaré rats (*Thrichomys apereoides*) (Monteiro et al., 2003), where individuals were sampled along an environmental gradient and populations from arid environments had larger coronoid processes, larger jugals, and wider snouts compared to non‐arid populations. This is thought to be related to bite force and linked to the vegetation type of the region (Monteiro et al., 2003). As such, bite force has a strong influence on muscle development, even within a species' lifetime. Evidence is emerging that bite force may also have wider implications regarding social structure and reproductive success, and thus increased selection pressure (Kraus et al., 2022). The greater skull depth observed in the arid populations suggests a greater action of the chisel digging method, likely required due to the hardness of the soil (McIntosh & Cox, 2016b). Furthermore, the different biomes of the arid and mesic regions consist of different vegetation (Wright & Samways, 1996). Arid populations of common mole‐rats will be feeding on desert geophytes that are tougher, and with thicker skins to reduce water loss in arid environments (Robb et al., 2016), and thus will require more chewing to break down. This increased need for stronger masticatory action in arid regions could also explain the larger inter‐shoulder widths and larger attachments for maxillary musculature in these populations. The clustering of the intermediate population for both body and skull measurements shows that there are more similarities in the diet and soil types of Klawer to the mesic population than to that of the arid. Future work could explore the mass of masticatory muscles, such as those of the masseteric complex and temporal region. This would allow for comparison of the relative muscle mass between the arid and mesic populations in relation to the use of incisors. Magnetic Resonance Microscopy may enable imaging and quantification of muscle volume, without the need for excision (Driehuys et al., 2008). Paired with data on soil hardness, this study would shed light on the direct effects of soil type on skull morphology of common mole‐rats.

## 5 CONCLUSION

Aridity embraces a series of habitats that covers a great deal of the Earth's surface and, thus, it is important to understand how environmental factors directly influence morphological traits across populations of the same species, along an environmental gradient. We have found evidence for extensive variation in body and skull morphology of populations of *C. h. hottentotus* found in arid and mesic populations. The separate clustering of mesic and arid individuals with different morphologies suggests the environment does play a significant role in the morphological variation seen in populations of *C. h. hottentotus*. These differences are counter to what is expected in mammals and indicate arid and mesic mole‐rat populations show unique adaptations in response to the food source variation and soil composition of the different biomes. This study contributes to a better understanding of the intra‐specific morphology of mole‐rats distributed along an aridity gradient, and skull morphology as an adaptive response to aridity and food distribution. We can also gain an insight into how subterranean species can change their highly specified subterranean morphology to cope with climatic differences. This can help to infer the level of adaptive morphological specificity within species and highlight the challenges they may face in changing climates and whether it is possible for a species to change its morphology, and rapidly enough, to cope with climate changes.

## AUTHOR CONTRIBUTIONS

**Hana N. Merchant:** Conceptualization (equal); data curation (equal); formal analysis (equal); funding acquisition (equal); investigation (equal); methodology (equal); project administration (equal); software (equal); visualization (equal); writing – original draft (equal); writing – review and editing (equal). **Steven J. Portugal:** Conceptualization (equal); methodology (equal); project administration (equal); resources (equal); software (equal); supervision (equal); validation (equal); writing – review and editing (equal). **Nigel C. Bennett:** Conceptualization (equal); data curation (equal); supervision (equal); validation (equal); writing – review and editing (equal). **Chris G. Faulkes:** Conceptualization (equal); project administration (equal); supervision (equal); validation (equal); writing – review and editing (equal). **Andries K. Janse van Vuuren:** Data curation (equal); validation (equal). **James Bowen:** Methodology (equal); resources (equal); software (equal); validation (equal); writing – review and editing (equal). **Daniel W. Hart:** Conceptualization (equal); data curation (equal); investigation (equal); supervision (equal); validation (equal); writing – review and editing (equal).

## CONFLICTS OF INTEREST STATEMENT

We have no competing interests.

## Supporting information


Data S1.

Data S2.

Data S3.

Table S1.

## Data Availability

All data are available as an electronic File S1.
