# Supplementary material for: New insights into morphological adaptation in common mole‐rats (Cryptomys hottentotus hottentotus) along an aridity gradient
Source: Ecol Evol. 2024 Apr 21;14(4):e11301. doi: 10.1002/ece3.11301 (PMC11033624; doi:10.1002/ece3.11301)
Supplement: Supplementary file 3 — Data S3. [file ECE3-14-e11301-s001.docx]

**Supplementary Information**

**Table S1.** Full list of samples used in this study with sample ID, population origin and colony, followed by sex and age class (Bennett et al., 1990). All samples used in this study were non-breeding individuals. The use of each sample for each of the three sections of data is marked with a Y (yes) or N (no) and are listed as organs (organs mass data), body (external body measurements), skulls (3D skull morphometrics) and fur (fur colour and thickness data). Samples were not used in some data sets due to data not being available to collect or in the case of skull samples, damage to the specimens making 3D visualisations unusable for analysis.

***SEE EXCEL FILE: Table S1***

**Table S2**. Table listing all tissues extracted during dissections, in order of extraction. Organs were divided and stored in different solutions in accordance with the needs of various studies being carried out.

| **Tissue** | **Storage** |
| --- | --- |
| **Heart 1** | RNA-Later |
| **Heart 2** | -80 |
| **Lung 1** | RNA-Later |
| **Lung 2** | -80 |
| **Liver 1** | -80 |
| **Liver 2** | RNA-Later |
| **Liver 3** | -80 |
| **Liver 4** | Formalin |
| **Spleen 1** | -80 |
| **Spleen 2** | Formalin |
| **Spleen 3** | RNA-Later |
| **Kidney 1** | -80 |
| **Kidney 2** | Formalin |
| **Whole blood** |  |
| **Blood serum** | -80 |
| **Blood plasma** | -80 |
| **Gonads** | Formalin |
| **GIT** | Formalin |
| **Eyes** | Formalin |
| **Skin** | RNA-Later |
| **Skeletal muscle 1** | 70% ethanol |
| **Skeletal muscle 2** | -80 |
| **Skeletal muscle 3** | -80 |
| **Skeletal muscle 4** | -80 |
| **Skeletal muscle 5** | RNA-Later |
| **Head** | -20 |
| **Body** | -20 |


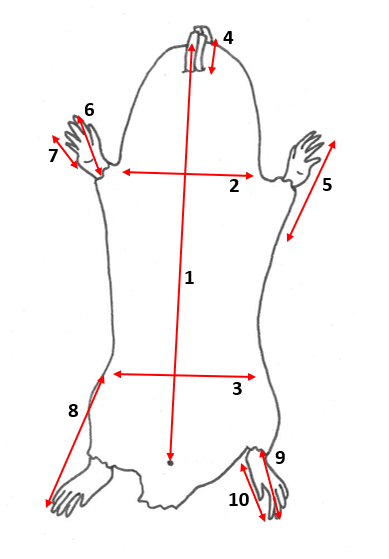


**Figure S1**. Outline drawing of *C. h. hottentotus* individual from a ventral view with 10 measurements shown. Numbers denoting measurements taken correspond with Table 1.

**Supplementary Information 1.** Methodological details for measurements of fur absorbance, reflectance and thickness.

A 10mm-by-10mm patch of fur was cut from the left thigh of the bodies. Fur absorbance and reflectance were measured using Ocean Optics spectrophotometer (Ocean Optics USB2000, Oxford, UK) measuring between 329nm – 1000nm. Prior to taking measurements the device was calibrated using a WS-1 Diffuse reflectance standard both with and without a 215-light source (HL-2000 Tungsten Halogen Light Source, Ocean Optics, Oxford, UK). The reflectance probe and light source were held at a standardised distance in a probe holder at a 90° angle (i.e., perpendicular) from to the sample for absorbance, and at a 45° angle for reflectance (McGraw, 2004). Five repeats per fur sample were taken by placing the probe on the patch of fur to provide full coverage of the fur from base to tip across the whole patch. Absorbance and reflectance were used to quantify fur colour. Following this, to measure thickness, each piece of fur was weighed using an electronic precision analytical weighing balance (BIOBASE, BP1003B), to the nearest mg. An assumed thickness was based on the mass (in mg) of 100 hairs, using a microbalance (Mettler Toledo, Balance XPR2U), across 5 individuals per population, which was not found to be significantly different among populations (ANOVA: F = 0.364, df = 4, p = 0.83).

**Supplementary Information 2.** Methodological details of skull extraction.

Once body measurements were complete, the heads were removed from the bodies of sixty individuals (number of individuals used in corresponding respirometry study) using scissors, at the base of the skull below the Atlas and Axis bones, at the third cervical vertebra. Skin was pulled off and then as much muscle was removed from the skull as possible using dissection blades and scissors. The heads were left to dry overnight and subsequently placed in cloth bags and boiled in water for two hours. The remaining soft tissue was removed as much as possible. The heads were then placed back in boiling water for a further hour. The remaining flesh was separated from the surface of the skull. Skulls were left at room temperature to dry for a few hours before being stored in a -20°C freezer overnight. The final pieces of tissue were subsequently peeled off following freezing leaving clean skulls and disarticulated lower jaws.


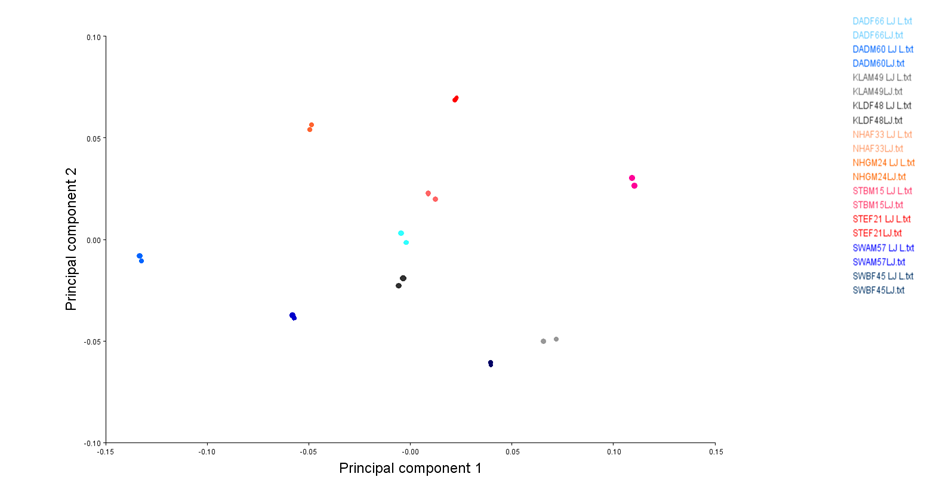


**DA66-L**

**DA66-R**

**DA60-L**

**DA60-R**

**KL49-L**

**KL49-R**

**KL48-L**

**KL48-R**

**NH33-L**

**NH33-R**

**NH24-L**

**NH24-R**

**ST15-L**

**ST15-R**

**ST21-L**

**ST21-R**

**SW57-L**

**SW57-R**

**SW45-L**

**SW45-R**

**Figure S2.** PCA plot of left and right sides of the jaw for 10 individuals (2 per population) from each of the 5 populations, Steinkopf (ST), No Heep (NH), Klawer (KL), Darling (DA) and Somerset West (SW). This preliminary analysis was carried out in MorphoJ Version 2.0 (Klingenberg, 2011) to assess the magnitude of symmetry in the lower jaw, which was found to be high.

**Table S3.** Eigenvalues for PCA shown in Figure 2. The first three dimensions explain >70% of the variance, demonstrating that the differences shown are sufficiently explained by the data.

|  | **eigenvalue** | **variance percent** | **cumulative variance percent** |
| --- | --- | --- | --- |
| **Dim 1** | 2.17 | 43.31 | 43.31 |
| **Dim 2** | 1.17 | 23.39 | 66.70 |
| **Dim 3** | 0.76 | 15.24 | 81.94 |
| **Dim 4** | 0.49 | 9.96 | 91.9 |
| **Dim 5** | 0.4 | 8.09 | 100 |

**Figure S3.** Contribution plot showing the amount of representation of each variable in PC1 and 2 for the PCA. Liver, lungs and kidney mass are the top contributors.


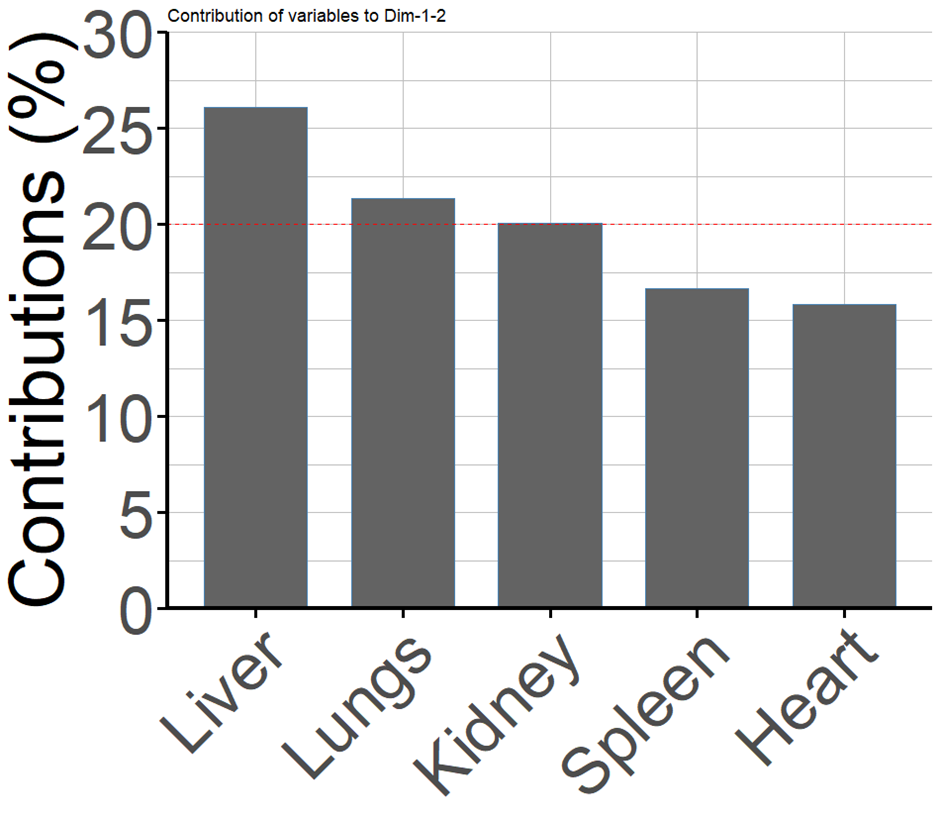


**Figure S4.** Principal component analysis based on organ masses of 71 individuals of *C. h. hottentotus* for males and females. The distribution is not influenced by sex differences. Confidence ellipses are shaded according to population colour and define the region containing 95% of the samples drawn from the underlying Gaussian distribution. Contributions of each variable used in the PCA analysis are displayed using a gradient, blue indicating the highest contribution and red the lowest contribution.


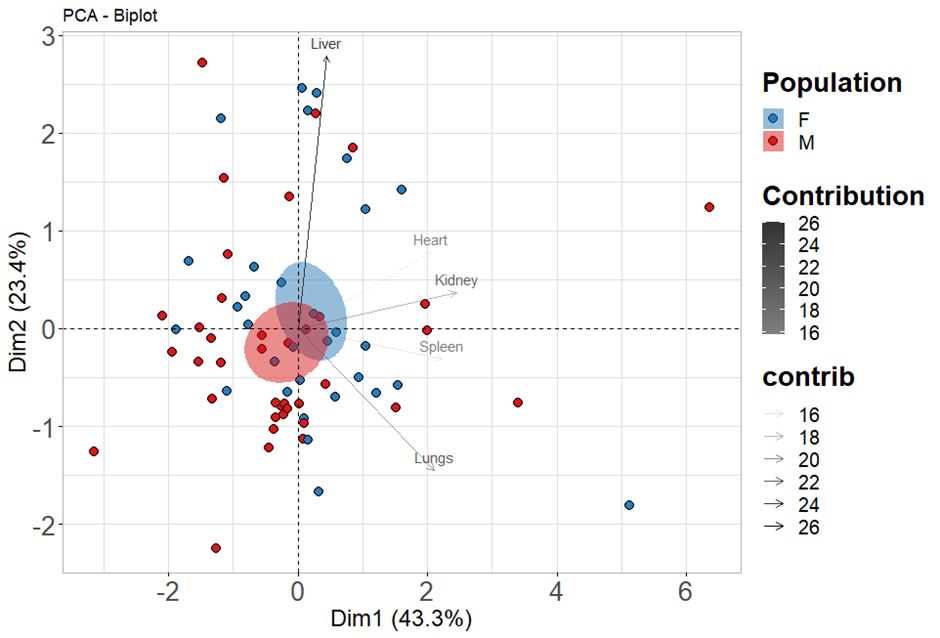


**Table S4.** Eigenvalues for PCA shown in Figure 3. The first three dimensions explain >70% of the variance, demonstrating that the differences shown are sufficiently explained by the data.

|  | **eigenvalue** | **variance percent** | **cumulative variance percent** |
| --- | --- | --- | --- |
| **Dim 1** | 4.62 | 41.98 | 41.98 |
| **Dim 2** | 2.31 | 21.03 | 63.01 |
| **Dim 3** | 0.83 | 7.57 | 70.58 |
| **Dim 4** | 0.77 | 6.97 | 77.56 |
| **Dim 5** | 0.64 | 5.84 | 83.4 |
| **Dim 6** | 0.49 | 4.42 | 87.82 |
| **Dim 7** | 0.4 | 3.67 | 91.49 |
| **Dim 8** | 0.33 | 3.02 | 94.51 |
| **Dim 9** | 0.27 | 2.44 | 96.95 |
| **Dim 10** | 0.2 | 1.85 | 98.8 |
| **Dim 11** | 0.13 | 1.2 | 100 |

**Figure S5.** Contribution plot showing the amount of representation of each variable in PC1 and 2 for the PCA. Shoulder width, body mass and measures of the fore limb are the top contributors.


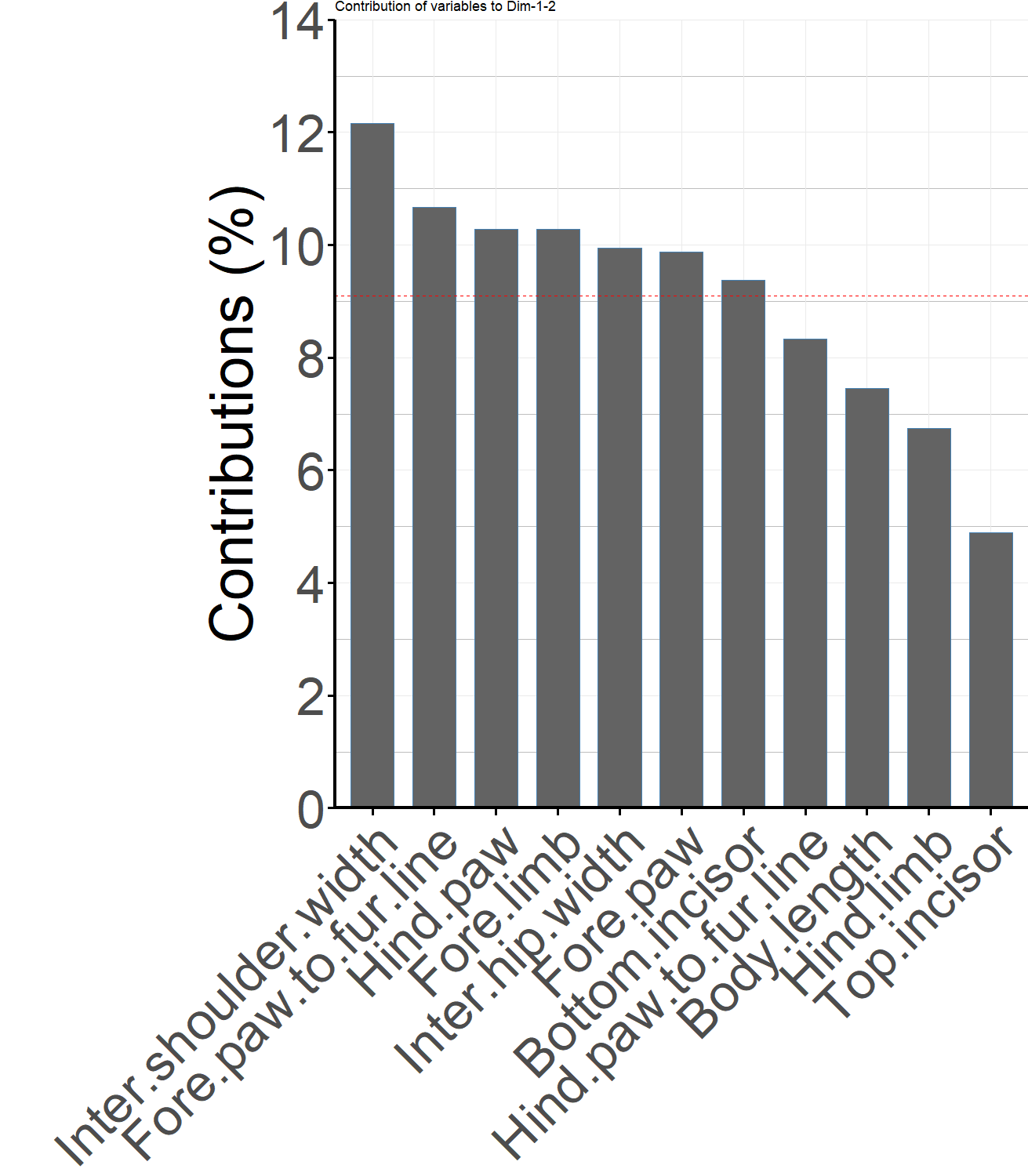

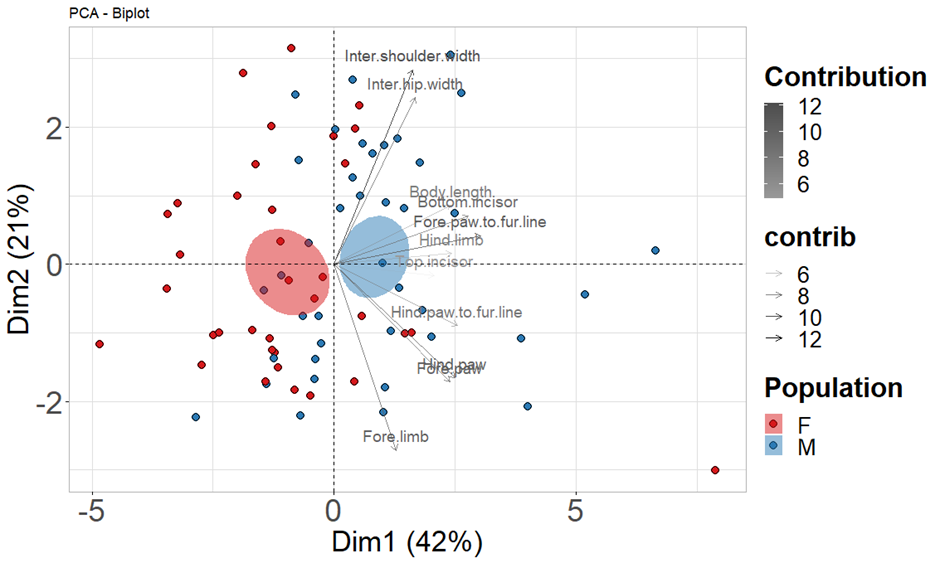

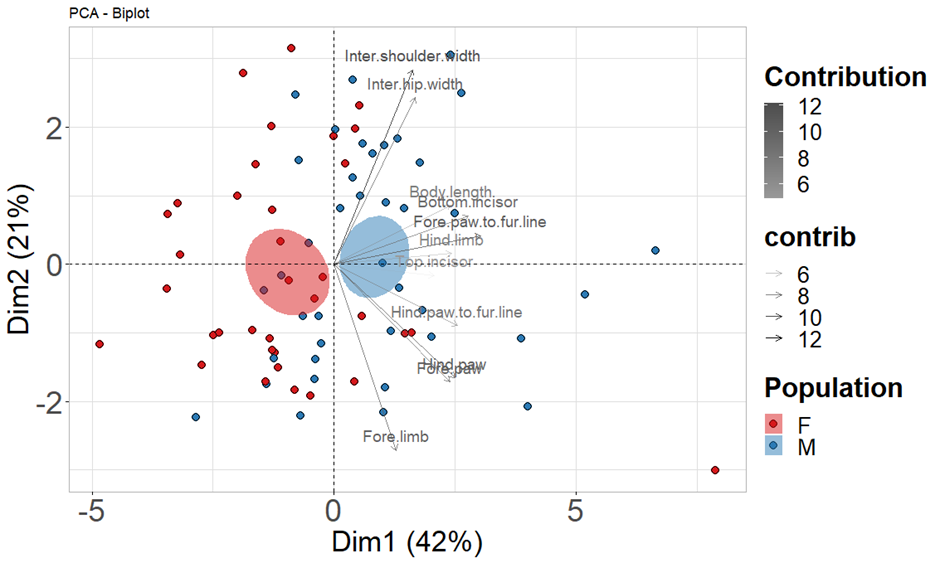

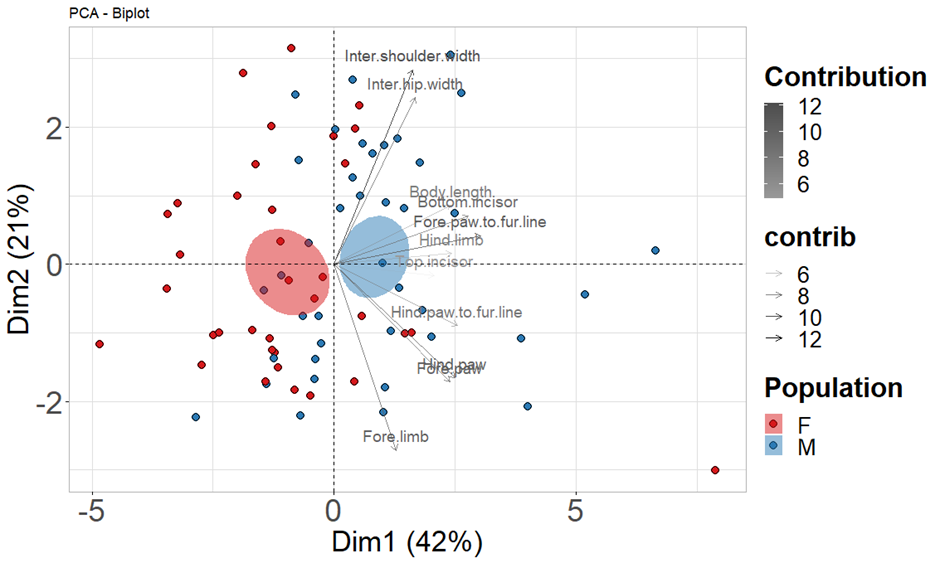


**Figure S6.** Principal component analysis based on body measurements of 75 individuals of *C. h. hottentotus* for males and females. The distribution is not influenced by sex differences. Confidence ellipses are shaded according to population colour and define the region containing 95% of the samples drawn from the underlying Gaussian distribution. Contributions of each variable used in the PCA analysis are displayed using a gradient, blue indicating the highest contribution and red the lowest contribution.

**Table S5.** Eigenvalues for PCA of the crania shown in Figure 5a. The first three dimensions explain 65.23% of the variance, demonstrating that the differences shown are not sufficiently explained by the data.

|  | **eigenvalue** | **variance percent** | **cumulative variance percent** |
| --- | --- | --- | --- |
| **Dim 1** | 20.23 | 25.61 | 25.61 |
| **Dim 2** | 17.55 | 22.21 | 47.82 |
| **Dim 3** | 13.76 | 17.41 | 65.23 |
| **Dim 4** | 9.74 | 12.33 | 77.56 |
| **Dim 5** | 7.22 | 9.14 | 86.71 |
| **Dim 6** | 2.66 | 3.37 | 90.1 |
| **Dim 7** | 1.83 | 2.32 | 92.4 |
| **Dim 8** | 0.99 | 1.26 | 93.65 |
| **Dim 9** | 0.79 | 1.01 | 94.66 |
| **Dim 10** | 0.68 | 0.87 | 95.52 |
| **Dim 11** | 0.54 | 0.68 | 96.2 |
| **Dim 12** | 0.43 | 0.55 | 96.75 |
| **Dim 13** | 0.39 | 0.49 | 97.24 |
| **Dim 14** | 0.31 | 0.39 | 97.62 |
| **Dim 15** | 0.3 | 0.37 | 97.99 |
| **Dim 16** | 0.24 | 0.3 | 98.3 |
| **Dim 17** | 0.23 | 0.29 | 98.59 |
| **Dim 18** | 0.2 | 0.26 | 98.85 |
| **Dim 19** | 0.15 | 0.2 | 99.04 |
| **Dim 20** | 0.1 | 0.12 | 99.17 |
| **Dim 21** | 0.1 | 0.12 | 99.29 |
| **Dim 22** | 0.08 | 0.11 | 99.39 |
| **Dim 23** | 0.07 | 0.09 | 99.49 |
| **Dim 24** | 0.06 | 0.07 | 99.56 |
| **Dim 25** | 0.05 | 0.06 | 99.62 |
| **Dim 26** | 0.04 | 0.05 | 99.67 |
| **Dim 27** | 0.03 | 0.04 | 99.71 |
| **Dim 28** | 0.03 | 0.04 | 99.75 |
| **Dim 29** | 0.02 | 0.04 | 99.79 |
| **Dim 30** | 0.03 | 0.03 | 99.82 |
| **Dim 31** | 0.02 | 0.02 | 99.85 |
| **Dim 32** | 0.02 | 0.02 | 99.87 |
| **Dim 33** | 0.02 | 0.02 | 99.89 |
| **Dim 34** | 0.02 | 0.02 | 99.91 |
| **Dim 35** | 0.01 | 0.02 | 99.93 |
| **Dim 36** | 0.01 | 0.01 | 99.94 |
| **Dim 37** | 0.01 | 0.01 | 99.95 |
| **Dim 38** | 0.01 | 0.01 | 99.96 |
| **Dim 39** | 0.01 | 0.009 | 99.97 |
| **Dim 40** | 0.01 | 0.007 | 99.98 |
| **Dim 41** | 0.004 | 0.006 | 99.98 |
| **Dim 42** | 0.004 | 0.005 | 99.99 |
| **Dim 43** | 0.004 | 0.004 | 99.99 |
| **Dim 44** | 0.003 | 0.004 | 99.99 |
| **Dim 45** | 0.002 | 0.002 | 100 |

**Table S6.** Eigenvalues for PCA of the lower jaw shown in Figure 5b. The first three dimensions explain 65.97% of the variance, demonstrating that the differences shown are not sufficiently explained by the data.

|  | **eigenvalue** | **variance percent** | **cumulative variance percent** |
| --- | --- | --- | --- |
| **Dim 1** | 6.28 | 25.13 | 25.13 |
| **Dim 2** | 5.27 | 21.07 | 46.2 |
| **Dim 3** | 4.94 | 19.77 | 65.97 |
| **Dim 4** | 3.07 | 12.29 | 78.26 |
| **Dim 5** | 1.97 | 7.9 | 86.16 |
| **Dim 6** | 1.31 | 5.24 | 91.4 |
| **Dim 7** | 0.91 | 3.63 | 95.03 |
| **Dim 8** | 0.39 | 1.58 | 96.61 |
| **Dim 9** | 0.24 | 0.97 | 97.58 |
| **Dim 10** | 0.14 | 0.54 | 98.12 |
| **Dim 11** | 0.1 | 0.42 | 98.54 |
| **Dim 12** | 0.1 | 0.42 | 98.94 |
| **Dim 13** | 0.07 | 0.29 | 99.24 |
| **Dim 14** | 0.04 | 0.15 | 99.39 |
| **Dim 15** | 0.04 | 0.14 | 99.53 |
| **Dim 16** | 0.03 | 0.12 | 99.65 |
| **Dim 17** | 0.02 | 0.09 | 99.74 |
| **Dim 18** | 0.02 | 0.07 | 99.81 |
| **Dim 19** | 0.01 | 0.05 | 99.86 |
| **Dim 20** | 0.01 | 0.04 | 99.9 |
| **Dim 21** | 0.01 | 0.03 | 99.93 |
| **Dim 22** | 0.01 | 0.03 | 99.96 |
| **Dim 23** | 0.005 | 0.02 | 99.98 |
| **Dim 24** | 0.003 | 0.01 | 99.99 |
| **Dim 25** | 0.002 | 0.01 | 100 |

a


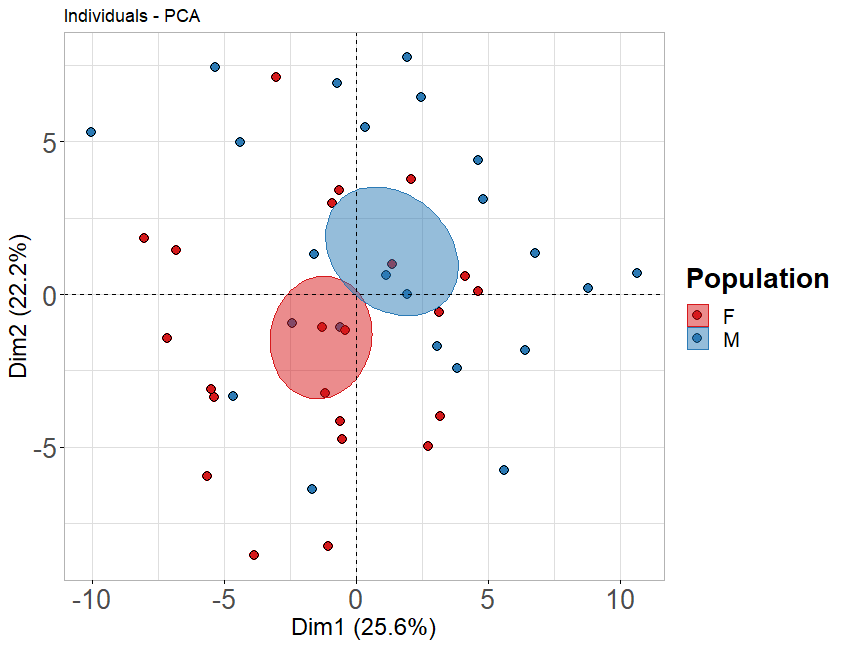


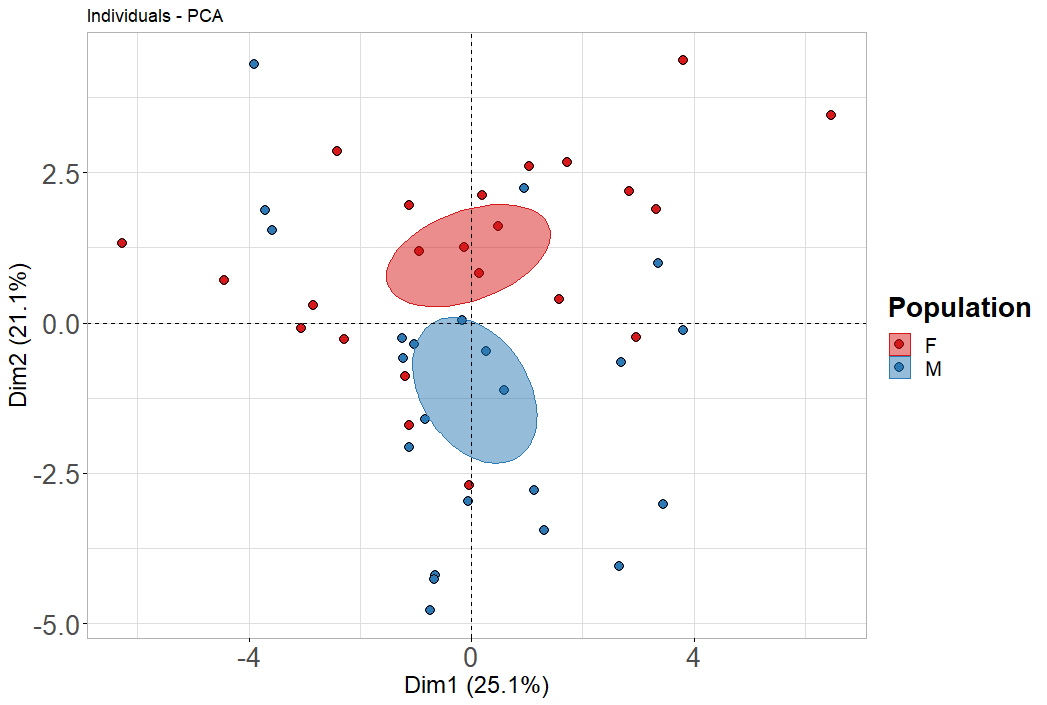


b

**Figure S7.** Principal component analysis based on the **a)** crania and **b)** lower jaw landmarks of 57 individuals of *C. h. hottentotus* for males and females. The distribution is not influenced by sex differences. Confidence ellipses are shaded according to population colour and define the region containing 95% of the samples drawn from the underlying Gaussian distribution.

a


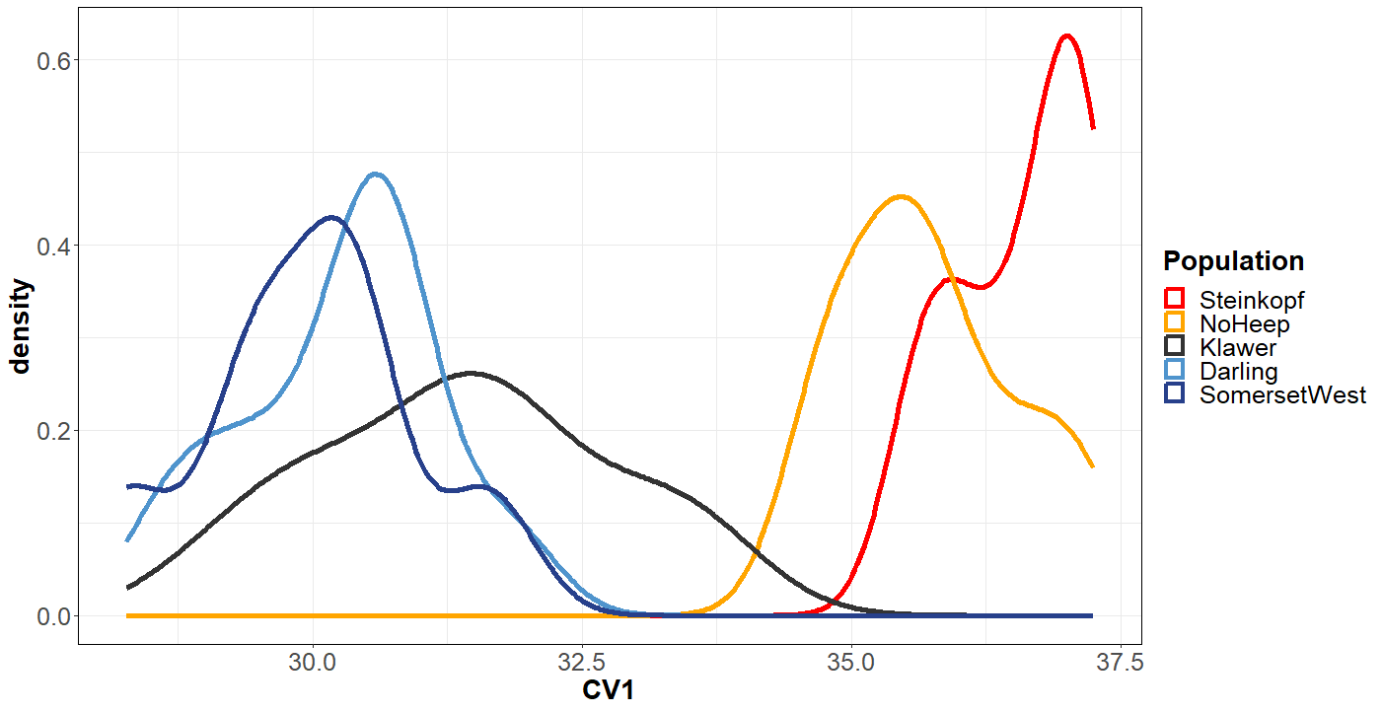


b


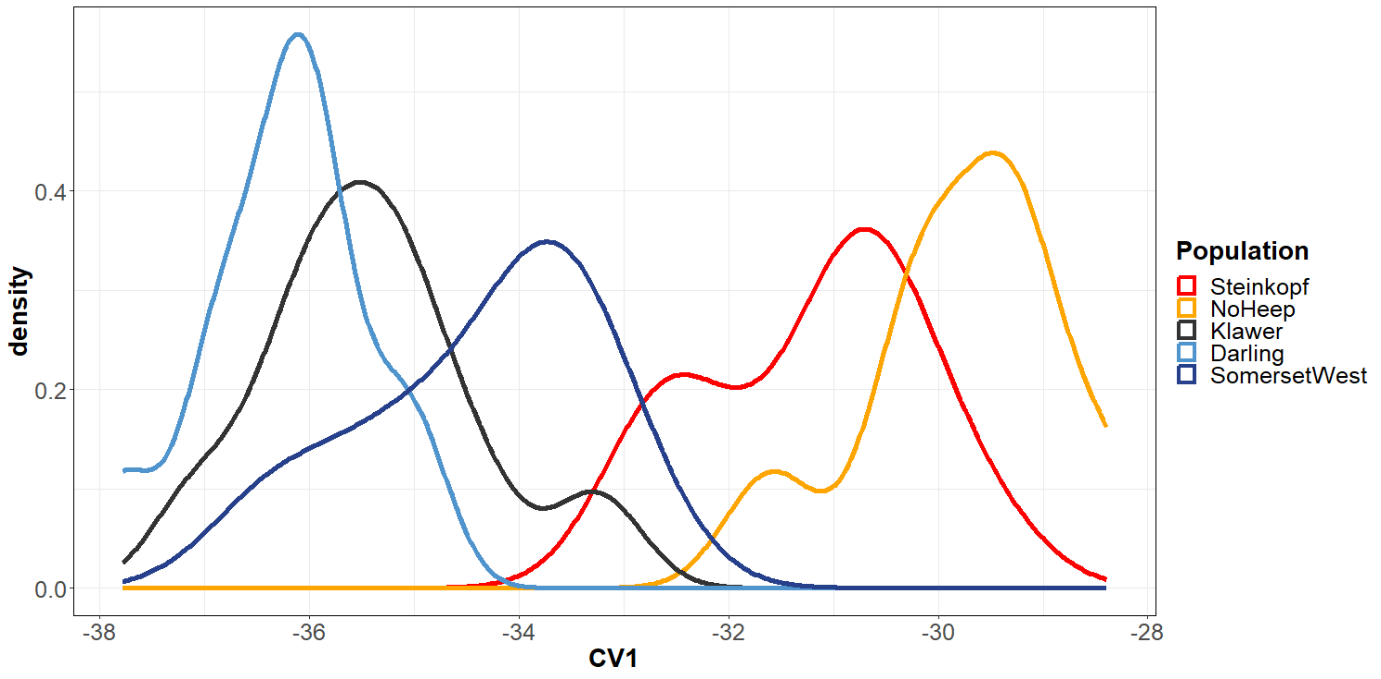


**Figure S8.** Density plot showing the distribution of individuals as explained by canonical variate 1 (CV1) for **a)** crania and **b)** lower jaw, derived from landmark data of 57 individuals of *C. h. hottentotus* across 5 populations, Steinkopf, No Heep, Klawer, Darling and Somerset West. The multimodal distribution demonstrates CV1 explains the variation between the mesic and arid populations.

**Somerset West**

**Steinkopf**

**No Heep**

**Klawer**

**Darling**


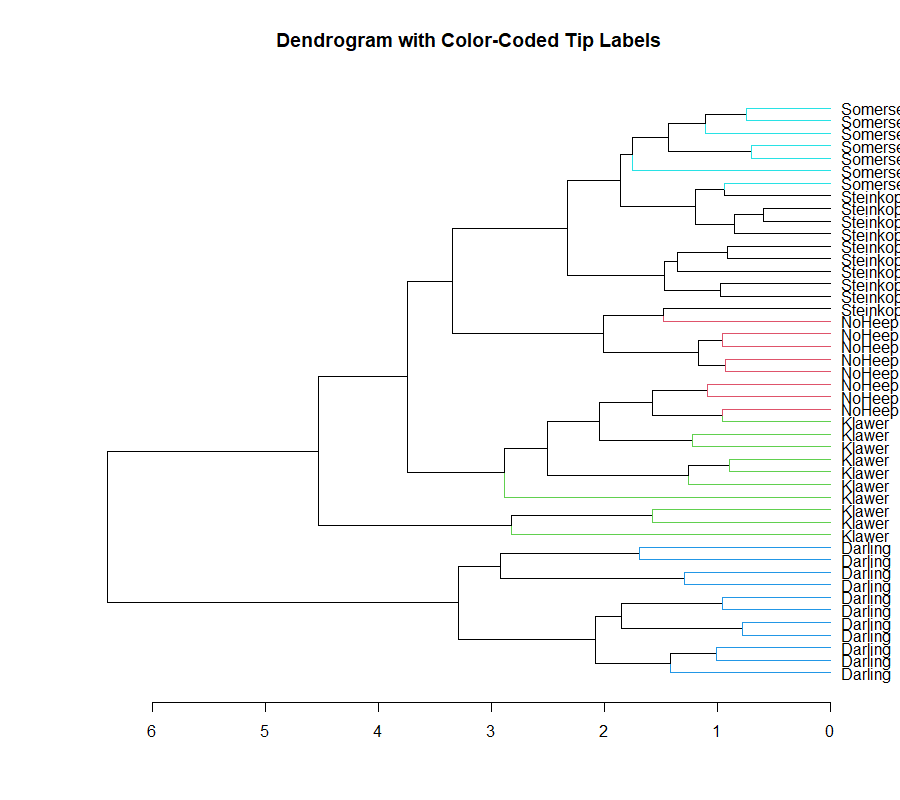


**Figure S9.** Phenogram based on skull (crania and lower jaw) morphometric data visualising the distance between the 57 samples of *C. h. hottentotus* across 5 populations, Steinkopf, No Heep, Klawer, Darling and Somerset West. Phenogram was created in R version 4.2.2 (R Core Team, 2021) using the packages cluster and dendextend.

**References**

Bennett, N.C., Jarvis, J.U.M. and Wallace, D.B., 1990. The relative age structure and body masses of complete wild‐captured colonies of two social mole‐rats, the common mole‐rat, *Cryptomys hottentotus hottentotus* and the Damaraland mole‐rat, *Cryptomys damarensis*. *Journal of Zoology*, 220(3), pp.469-485.

Klingenberg, C.P., 2011. MorphoJ: an integrated software package for geometric morphometrics. *Molecular Ecology Resources*, 11, pp.353-357.

McGraw, K.J., 2004. Multiple UV reflectance peaks in the iridescent neck feathers of pigeons. *Naturwissenschaften*, 91(3), pp.125-129.

R Core Team, 2021. R: A language and environment for statistical computing. R Foundation for Statistical Computing, Vienna, Austria. URL https://www.R-project.org/.
